# Integrated opposite charge grafting induced ionic-junction fiber

**DOI:** 10.1038/s41467-023-37884-0

**Published:** 2023-04-24

**Authors:** Yi Xing, Mingjie Zhou, Yueguang Si, Chi-Yuan Yang, Liang-Wen Feng, Qilin Wu, Fei Wang, Xiaomin Wang, Wei Huang, Yuhua Cheng, Ruilin Zhang, Xiaozheng Duan, Jun Liu, Ping Song, Hengda Sun, Hongzhi Wang, Jiayi Zhang, Su Jiang, Meifang Zhu, Gang Wang

**Affiliations:** 1 State Key Laboratory for Modification of Chemical Fibers and Polymer Materials, College of Materials Science and Engineering, Donghua University, 201620 Shanghai, China; 2 Department of Hand Surgery, Center for the Reconstruction of Limb Function, National Clinical Research Center for Aging and Medicine, Huashan Hospital; Department of Hand and Upper Extremity Surgery, Jing’an District Central Hospital; NHC Key Laboratory of Hand Reconstruction, Shanghai Key Laboratory of Peripheral Nerve and Microsurgery, Institute of Hand Surgery, Fudan University, 200040 Shanghai, China; 3 State Key Laboratory of Medical Neurobiology, MOE Frontiers Center for Brain Science, Institute of Brain Science, Department of Ophthalmology, Zhongshan Hospital, Fudan University, 200032 Shanghai, China; 4 Laboratory of Organic Electronics, Department of Science and Technology, Linköping University, SE-601 74, Norrköping, Sweden; 5 College of Chemistry, Sichuan University, 610064 Chengdu, China; 6 School of Automation Engineering, University of Electronic Science and Technology of China, 611731 Chengdu, China; 7 State Key Laboratory of Polymer Physics and Chemistry, Changchun Institute of Applied Chemistry, Chinese Academy of Sciences, 130022 Changchun, Jilin China; 8 National Key Laboratory on Electromagnetic Environmental Effects and Eletro-optical Engineering, 210007 Nanjing, China

**Keywords:** Electronic devices, Design, synthesis and processing

## Abstract

The emergence of ionic-junction devices has attracted growing interests due to the potential of serving as signal transmission and translation media between electronic devices and biological systems using ions. Among them, fiber-shaped iontronics possesses a great advantage in implantable applications owing to the unique one-dimensional geometry. However, fabricating stable ionic-junction on curved surfaces remains a challenge. Here, we developed a polyelectrolyte based ionic-junction fiber via an integrated opposite charge grafting method capable of large-scale continuous fabrication. The ionic-junction fibers can be integrated into functions such as ionic diodes and ionic bipolar junction transistors, where rectification and switching of input signals are implemented. Moreover, synaptic functionality has also been demonstrated by utilizing the fiber memory capacitance. The connection between the ionic-junction fiber and sciatic nerves of the mouse simulating end-to-side anastomosis is further performed to realize effective nerve signal conduction, verifying the capability for next-generation artificial neural pathways in implantable bioelectronics.

## Abstract (web summary)

Ionic-junction devices are difficult to integrate with fiber-shaped tissues like nerves and muscles for applications in implantable bioelectronics due to their large size and bulk structure. Authors realize here easy to implant fiber-shaped iontronics through an integrated opposite charge grafting process, enabling the construction of ionic logic gates and artificial neural pathways.

## Introduction

Computing and storage functions in typical electronic devices are realized based on the transport of electrons and/or holes, while these are mostly realized by ions in biological systems^1–3^. The difference in information carriers brings about the essential disparity between electronic devices and organisms, leading to a signal mismatch when designing bioelectronic interfaces for healthcare and human augmentation applications^4–7^. Growing interests have been attracted to overcome this barrier by employing intrinsic soft ionic conductor materials like hydrogels, polymeric gels, and mixed ionic-electronic conducting polymers in device design^1,8,9^. In recent years, newly emerged iontronics functions are achieved by combining ion transporting and electrical conductivity in organic materials, which could be utilized as a transducer between ionic and electronic circuits^8,10,11^. Reports showed that bipolar membranes and charged microchannels with opposite charges can be used to rectify and switch ion currents in biosensors^12^, logic gates^13^, and generators^14^. Suo and Hayward demonstrated that polyelectrolytes with anion or cation conductivity could be combined for ionic devices, including ionic diodes and ionic transistors, which hold logic functions similar to those in logic electronics and opened up the design of solid-state ion-electron hybrid devices^15^.

However, previously reported ionic-junction devices are all based on two-dimensional (2D) planar or three-dimensional (3D) bulk device structures. They are relatively large in size and challenging to connect with fiber-shaped biological organisms (e.g., nerves), thus prohibiting the applications for implanted bioelectronics^16^. Miniaturized fiber-based devices are similar to biological nerves in morphology, which favor deep penetration into various tissues with negligible damage and match well with them in terms of bending stiffness^17^. These properties would contribute to stable contact interfaces with biological tissues, allowing fiber devices to be implanted into the human body for accurate bioelectrical and biochemical information collection.

By combining the advantages of ionic polymer, including solution processability, ease of chemical modification, intrinsic stretchability, and biocompatibility, with one-dimensional (1D) fiber structure, fiber-shaped iontronics can be effectively integrated with natural biological tissue, leading to better applicability for disease diagnosis, medical treatment, human enhancement, and so on^18–21^. The electric double layer (EDL) formed at the solid-electrolyte interface is the main driving force for the operation of ionic devices. However, the fabrication of nanometer-scale electron-ion transport pathways in the EDL interface on the surface of 1D logic devices is constrained owing to the unique one-dimensional geometry. This required sophisticated design with an ordered arrangement and precise patterning of multi-material components, but popular high-precision patterning methods such as photolithography and nanoimprinting are not easily applicable on a curved surface, making 1D devices difficult to achieve satisfactory performance^22,23^. Moreover, the continuous and large-scale production of ionic-junction fibers with multilayer interfaces remains challenging. Traditional spinning methods (wet spinning, melt spinning, electrostatic spinning, and thermal drawing) cannot precisely control functional layer and interface properties due to strict control requirements (varying operating environments for different functional materials, diffusion coefficient, the surface tension of the solution, tensile force and shear force at the needle, etc.) during multi-spindle needle spinning^24^. Multilayer coating technology, based on electrostatic self-assembly, would be a preferred method for the integrated fabrication of fiber-shaped iontronics owing to its capability to precisely regulate the characteristics of carriers (number and migration rate) on the functional interface at the molecular level. However, coatings on 1D fiber surfaces tend to be highly non-uniform compared to 2D planes due to higher surface tension and unintentional flow under gravity^25^. This will result in inconsistent rectification performance, even failure, and accidental short circuits in the device, which limits the wide application of fiber-shaped iontronics. Therefore, it is still challenging to develop a method capable of continuously assembling fiber-shaped iontronics with a uniform and robust EDL interface^26^.

In this work, the concept of integrated opposite charge grafting (IOCG) of the commercially available polymer was demonstrated to fabricate ionic-junction fibers. Poly(styrene-b-(ethylene-co-butylene)-b-styrene) (SEBS) was selected as the polymer backbone. Na^+^ and Cl^−^ were selected as free-moving ions, which can satisfy the effective ion transport of the device at the internal temperature of the human body. Based on the operating mechanism that coupling between ions and electrons is driven by the EDL, circuit elements such as ionic diodes and ionic bipolar junction transistors (IBJTs), as well as the essential functions of neurons, including synaptic characteristics and neural signal transmission were demonstrated on the same fiber. The rectification ratio of the prepared fiber-shaped ionic diode reaches 30 and the switching ratio of the IBJTs is as high as 50, which is comparable to the planar counterparts. Enabled by the circuit design, two types of ionic-junction logic gates (“AND” gate and “OR” gate) were successfully constructed, performing logic operations on binary inputs. Moreover, the apparent synaptic function was also observed by applying a pre-pulse signal to the transistor with the presence of memory capacitance. To exhibit possible applications of the devices in organisms, end-to-side anastomosis of ionic-junction fibers with the sciatic nerve is realized as a neural pathway in the hindlimb of a mouse for neural stimulation in vivo, which preliminarily verifies possible clinical application in the future. It is anticipated that the method for an integrated preparation of ionic-junction fiber and its logical responsiveness, synaptic plasticity, and biocompatibility will open new avenues for integrating biological neural networks and combining the human-machine interface.

## Results

### Integrated fabrication of ionic-junction fiber

**Fig. 1:**
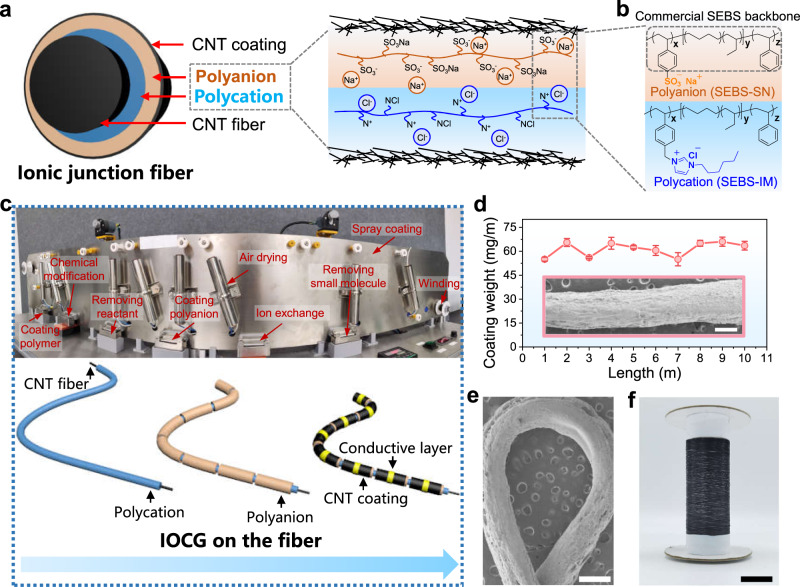
Integrated fabrication and structural characterization of ionic-junction fibers. **a** Illustration of the cross-sectional structure of ionic-junction fibers, along with the schematic of mobile Cl^−^ and Na^+^ ions diffusing in opposite directions by external voltage bias. **b** Molecular structures of polycations (SEBS-IM, SEBS grafting 3-hexylimidazolium groups) and polyanions (SEBS-SN, SEBS grafting sodium sulfonate groups) based on SEBS backbone. **c** Photograph of integrated fabrication line and schematic of molding process of ionic-junction fibers. **d** Maintaining loading weight density per meter of ionic-junction fiber with increasing fiber length (Inset, scanning electron microscope (SEM) image of an ionic-junction fiber, representative of three experiments, scale bar = 500 μm). **e** SEM image of a bent ionic-junction fiber (curvature radius of 1.5 mm) with no obvious peeling of the carbon nanotube (CNT) coating layer, representative of 3 experiments, scale bar = 500 μm. **f** Reels of ionic-junction fibers. Scale bar = 2 cm. Source data are provided as a Source Data file.

Figure 1a presents a cross-sectional schematic of an ionic-junction fiber consisting of a core carbon nanotube (CNT) fiber with high conductivity (1 × 10^5^-2 × 10^5^ S/m), polycationic and polyanionic functional layer, and CNT sheath. Polycation/polyanion heterojunction led to the formation of an ionic double layer (IDL), which mimics the depletion region in a semiconducting P-N junction^15,27^, as shown in the diagram on the right in Fig. 1a. Compared to the coating on planar substrates, however, it is worth noting that coatings of polyelectrolytes on curved fiber surfaces tend to be uneven. The challenges can be attributed to that the slurry experiences high surface tension resulting from additional Laplace pressure^28–30^. Therefore, it is very challenging to obtain a robust, high-loading, and yet uniform coating of polycation and polyanion around long CNT fiber that ranges within a hundred-micrometer scale in diameter^25^. In our work, the concept of IOCG is proposed to design fiber-shaped ionic-junction devices, employing the modification of commercially available polymer SEBS. Polycations (SEBS-IM, SEBS grafting 3-hexylimidazolium groups) and polyanions (SEBS-SN, SEBS grafting sodium sulfonate groups) are designed onto SEBS backbone, with synthesizing process in Supplementary Figs. 1 and  2. Cl^−^ and Na^+^ were chosen as the counter ions, respectively, due to their relatively high mobilities compared to other organic counterparts and the fact of being among the most common anions and cations in the human body^31^. Figure 1b demonstrates the molecular structures of polycations and polyanions based on the SEBS backbone in IDL heterojunctions. The differential scanning calorimetry (DSC) results showed that the *T*_g_ of both polyelectrolytes is close to 37 °C, which enables selective ion conduction of these polyelectrolytes in the human body (Supplementary Fig. 3). Other detailed experimental characterizations like attenuated total reflection Flourier transformed infrared spectroscopy (ATR-FTIR), surface energy (contact angle (CA)) and thermogravimetric analysis (TGA) of two polyelectrolytes are described in the Supplementary Information (see Supplementary Figs. 4, 5), further proving that the introduction of ionic liquid changed the hydrophilicity of the polycation and reduced the degree of water loss in air.

The continuously integrated polyelectrolyte grafting equipment was designed to produce the ionic conducting fibers at a kilometer scale (Fig. 1c and Supplementary Movie 1). Supplementary Fig. 6 shows the fabrication process of the ionic-junction fiber implemented through loading two polyelectrolytes with oppositely charged on a thin CNT fiber (diameter of 60-80 μm) via a layer-by-layer coating method. Particularly, the quaternization reaction and ion exchange steps required heating to increase the rate and extent of the reaction. Some heating drums were utilized in the coating process to dry and anneal each layer of materials after coating. Finally, pre-prepared CNT slurries were deposited on the outer of the polyanion to serve as a capacitor layer by spraying coating. The schematic illustration in Fig. 1c presents the molding process from the CNT fiber to the target fiber device. As shown in Supplementary Fig. 7, uniform and transparent polyelectrolytes were loaded on CNT fiber firmly layer by layer (the thickness of polycation is 180-220 μm and polyanion is 140-160 μm). Supplementary Fig. 8 showed the scanning electron microscope (SEM) image of the surface of CNT fiber (core) and CNT coating (sheath). In addition, the coating weight of the fiber device was nearly unchanged along the investigated 10-m-long fiber (Fig. 1d), suggesting the uniformity of polyelectrolyte in a continuous process. Additionally, bent fibers showed no obvious peeling or cracking of functional materials (Fig. 1e). After continuous coating preparation, ionic-junction fiber as long as 1000 m was fabricated continuously and collected onto several bobbins at a winding speed of 10 cm/min owing to the intrinsic flexibility of the organic components and fiber-shaped device geometry (Fig. 1f). Scale-up of the fiber-shaped logic devices process through this integrated coating preparation to produce kilometer-long ionic-junction fibers is easily envisioned.

### Rectification characteristics of fiber-shaped ionic diodes

**Fig. 2:**
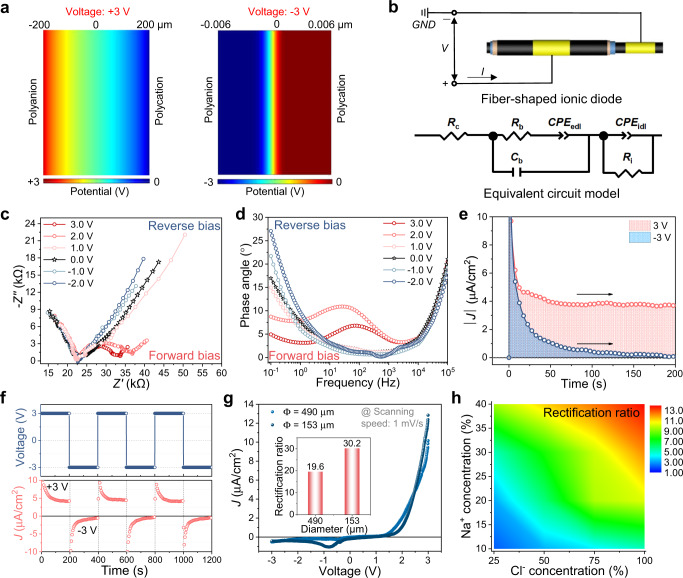
Ion rectification effect of fiber-shaped ionic diodes. **a** Calculated induced potential distribution profile along the thickness direction of fiber-shaped ionic diodes by applying ±3.0 V, respectively. The right side of the model is grounded. **b** Circuit diagram of a fiber-shaped ionic diode and the equivalent circuit model for a SEBS-IM/SEBS-SN junction under direct current (DC) bias of +3.0 V. *V* and *I* are the voltage and current through the external circuit of the ionic diode; *GND*, ground; *R*_c_, contact resistance; *R*_b_, a resistor reflects the drift of free ions; *C*_b_, a dielectric capacitance;* CPE*_edl_ describing the EDL capacitance and *CPE*_idl_ describing the ionic double layer (IDL) capacitance; *R*_i_, interfacial resistance. **c**, **d** Nyquist plot and Bode phase plot from alternating current (AC)-impedance measurements of the fiber-shaped ionic diode under DC biases. **e** The current density of the SEBS-IM/SEBS-SN junction under a forward bias of +3.0 V and a reverse bias of −3.0 V, applied at 0 s. **f** Rectification by SEBS-IM/SEBS-SN junctions under an alternating potential of ±3.0 V (applied voltage is plotted on the top, while the corresponding current is demonstrated on the bottom). **g** Current density as a function of applied bias for a fiber-shaped ionic diode with different diameters (Ø). Scanning rate is 1 mV/s (inset, rectification ratio of different fiber-shaped ionic diodes with different diameters). **h** Contour color-filled comparative maps of a trend of rectification ratio at different ion concentrations. Scanning rate is 10 mV/s. Source data are provided as a Source Data file.

The diode-like behavior of the polyelectrolyte heterojunction could be anticipated in the ideal situation when an electric field is pulling the mobile Na^+^ and Cl^−^ ions away from the interface, and the thus formed narrow depletion area would lead to a “cutoff” state to prohibit currents from going through (Supplementary Fig. 9)^15^. Figure 2a illustrates the potential distribution across the heterojunction interface at forward and reverse voltage, simulated with Poisson-Nernst-Planck model^32,33^. We could see under forward bias, the potential is relatively distributed even through the electric field direction, representing the “ON” state of the diode. But when a reverse bias is applied, the voltage dropped mostly across the nanometer scale narrow depletion region beside the interface, matching well with the situation in the diode “OFF” state. More information could be found in the Supplementary Information.

The corresponding fiber-shaped ionic diode was fabricated owing to forming the IDL heterojunctions on the interface between polycations and polyanions, as shown in Supplementary Fig. 10. As shown in Fig. 2b, an equivalent circuit model was first developed to model electrochemical behavior in the fiber-shaped ionic diode, containing elements representing the interfacial capacitance (*CPE*_idl_) and resistance (*R*_i_). *R*_i_ rapidly decreased as mobile ions accumulated at the interface to destroy the IDL with increasing forward bias, resulting in decreased low-frequency impedance (red line in Fig. 2c) as well as a prominent decrement in low-frequency peak corresponding to the IDL capacitance (Fig. 2d). Fitting curves and parameters of the circuit components in the equivalent circuit model were shown in Supplementary Fig. 11 and Supplementary Table 1. Moreover, no obvious redox reaction was expected at the interface of the carbon nanotube electrode in our device within the operation voltage window from −3.0 to 3.0 V (Supplementary Fig. 12). When applied a forward bias voltage of +3.0 V, as shown in Fig. 2e, there was no significant decay in the current density ( *J*), which proved that the diode was conducting, similar to a low-value resistor. In contrast, a reverse bias of −3.0 V yielded an obvious exponential decay of current over a time (*t*). Full *J*-*V* curves of all ion polyelectrolyte junctions (SEBS-IM/SEBS-IM, SEBS-SN/SEBS-SN, and SEBS-IM/SEBS-SN) summarized in Supplementary Fig. 13 show that SEBS-IM/SEBS-IM and SEBS-SN/SEBS-SN homojunction had ideal linear curves of analogical magnitude to the values from AC-impedance. On the contrary, an apparent asymmetrical *J*-*V* curve was observed for SEBS-IM/SEBS-SN heterojunctions. The current response of fiber-shaped ionic diodes exhibited hysteresis attributed to the low ionic mobility.

Exploiting this asymmetry, a rectifying solid-state fiber-shaped ionic diode was demonstrated in Fig. 2f. The rectification ratio ( *J*+/*J*−, where *J*+ and *J*− denote the current density at forward and reverse bias) of the fiber-shaped ionic diode (The diameter: 153 μm) at ±3.0 V can reach about 30 at 1 mV/s, even among the best of previously reported planar counterparts (Fig. 2g, Supplementary Fig.14)^34–36^. The current increase in forward bias for thinner devices is a consequence of smaller bulk-dependent impedance, while the backward current is more of interface limited. Estimated by an exponential fit of the current density versus time, the storage time and the transition time of the fiber-shaped ionic diode (diameter: 153 μm) were 8.7 and 18.2 s, respectively (Supplementary Fig. 15). The relatively long transient response time is due to the slow rate of ion migration in polyelectrolytes. Furthermore, the rectifying behavior of the diode in different scan rates from 1 mV/s to 1 V/s was evaluated using linear sweep voltammetry (Supplementary Fig. 16). Under forward bias, the IDL is destroyed once the bias exceeds the voltage threshold of 1.2 V (Supplementary Fig. 17). Moreover, contour color-filled comparative maps in Fig. 2h indicates that the rectification ratio of fiber-shaped ionic diodes could readily be tuned by adjusting the ionic concentration of Na^+^ and Cl^−^ by changing the grafting degrees of the SEBS, which makes such fiber-shaped ionic diodes pronouncedly attractive as components to control ionic currents in integrated circuits and implantable devices. A grafting ratio of 40% in SEBS-SN and 100% in SEBS-IM was chosen for device fabrication in the following sections unless otherwise stated.

### Demonstration of ionic-junction logic circuits

**Fig. 3:**
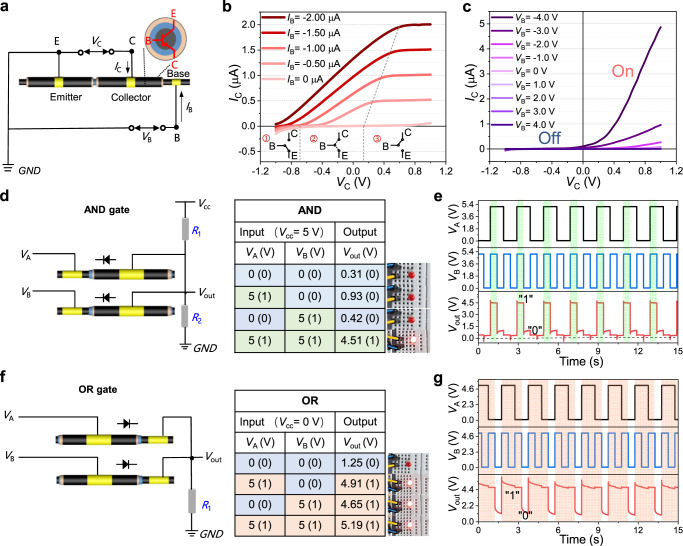
Demonstration of ionic-junction logic gates based on multiple fiber-shaped ionic diodes. **a** Device architecture and circuit diagram of a fiber-shaped IBJT connected in a common-emitter configuration. *V*_B_, the voltage of collector; *V*_C_, the voltage of collector; *I*_B_, the current of base; *I*_C_, the current of collector. **b** Output characteristic (*I*_C_-*V*_C_) curves as a function of input current (*I*_B_). **c** Input characteristic (*I*_C_-*V*_C_) curves as a function of input voltage (*V*_B_). **d** Schematic and truth table of an “AND” gate fabricated by two fiber-shaped ionic diodes. The square wave voltages (*V*_A_ and *V*_B_) were input through the core layer (CNT fiber) of two parallel fiber-shaped ionic diodes. The resistors *R*_1_ and *R*_2_ are 10 and 100 MΩ, respectively. *V*_A_, the voltage applied at terminal A; *V*_B_, the voltage applied at terminal B; *V*_CC_, power supply. **e** Experimental data of the input and output signals of the “AND” gate. **f** Schematic and truth table of an “OR” gate fabricated by two fiber-shaped ionic diodes, in which the square wave voltages (*V*_A_ and *V*_B_) were input through the sheath layer (CNT coating), where *R*_1_ is 100 MΩ. **g** Experimental data of the input and output signals of the “OR” logic gate. Source data are provided as a Source Data file.

After thoroughly characterizing the rectification capability of a single fiber-shaped ionic diode, we took advantage of the proposed fabrication process to integrate multiple diodes by parallel or in series to demonstrate ionic-junction logic circuits such as IBJTs and ionic-junction logic gates. A pnp-IBJT can be regarded as two pn-junctions sharing the same narrow base region (a lateral gap of <1 mm), where the emitter and collector are p-doped (SEBS-SN), and the base is n-doped (SEBS-IM). Therefore, a polyelectrolyte fiber-shaped IBJT based on fiber-shaped ionic diodes was demonstrated (Fig. 3a), and the corresponding preparation process was shown in Supplementary Fig. 18. The emitter (on the CNT coating) was grounded and supplied the emitter-base (on the CNT fiber) input current (*I*_B_), recording the emitter-collector output characteristic curves (*I*_C_-*V*_C_) based on cyclic potential sweeps from −1.0 to 1.0 V at 0.1 V/s (Fig. 3b). When *I*_B_= 0, either the E/B or C/B interface is always under reverse bias during the sweep of *V*_C_, resulting in a small *I*_C_ constrained by the IDL capacitance. In comparison, with negative *I*_B_, mobile anions in SEBS-IM are pushed to the interfaces between E/B and C/B, destroying the IDL and empowering a regime of linear resistive *I*_C_-*V*_C_ curves from *I*_B_= 0 μA to *I*_B_= −2.0 μA. The result was consistent with the curve trend of typical BJT behavior in Suo’s report^15^. Similarly, when a voltage is applied between the emitter and collector of a pnp-IBJT, the potential drop is divided over the emitter, junction, and collector (Fig. 3c). The ion concentration within the junction can be modulated by changing the base voltage (1.0 ~ −1.0 V) and in turn affecting the ionic current through the collector, which was consistent with the case for bipolar junction transistors previously reported^37,38^. The IBJT showed good transistor characteristics that have collector current on-off ratios up to 50.

In logic circuits, logic “0” is represented by voltages below a certain threshold level, while logic “1” is by voltages above another threshold level^39^. The first step toward more complex ionic circuits based on fiber-shaped ionic diodes and IBJTs is to implement some of these basic logic gates. Figure 3d illustrated the design of an “AND” gate formed by inputting the square wave voltage (*V*_A_ = *V*_B_ = 5 V) with the pre-set frequencies (*f*_A_ = 0.5 Hz, *f*_B_ = 1.0 Hz) through the core layer of two parallel fiber-shaped ionic diodes and the corresponding output voltage (*V*_out_) is received from the sheath layers, respectively. The truth table of the “AND” gate can be observed in Fig. 3d, where the light blue background and the bracketed binary number “0” represent the “low” status, while the light-yellow background and the bracketed binary number “1” represent the “high” status of the input/output signal. Consequently, the “high” status of the output signal is able to light a LED with a working voltage of 1.8 V. As shown in Fig. 3e, the input and output signals of the AND gate were experimentally measured, where the regions with light green background indicate the “high” status of the output.

Likewise, an ionic “OR” gate was assembled based on a converse connection (input from the sheath layer and output from the core layer) of two fiber-shaped ionic diodes. From the measured truth table in Fig. 3f, a precise “OR” operation was demonstrated, and the measured input/output voltage curves are shown in Fig. 3g. It is worth noting that there are minor peaks and drifts in each cycle, which can be attributed to the charging and discharging-induced delays of the pn-junctions and the *I*-*V* characteristic inconsistency between the two pn-junctions^38^. Additionally, logic gates are also tested with 50% and 90% duty cycle square wave at long-term (Supplementary Fig. 19). It is believed that the proposed fiber-shaped ionic-junction logic circuit may realize some new functions, including ion rectification as well as processing and computation of discrete signals in diverse promising applications, such as controlling delivery of ionic species, mimicking ionic-related biological systems and developing advanced iontronics intelligent devices^40^.

### Synaptic characteristics and switching mechanism of ionic-junction logic fiber

**Fig. 4:**
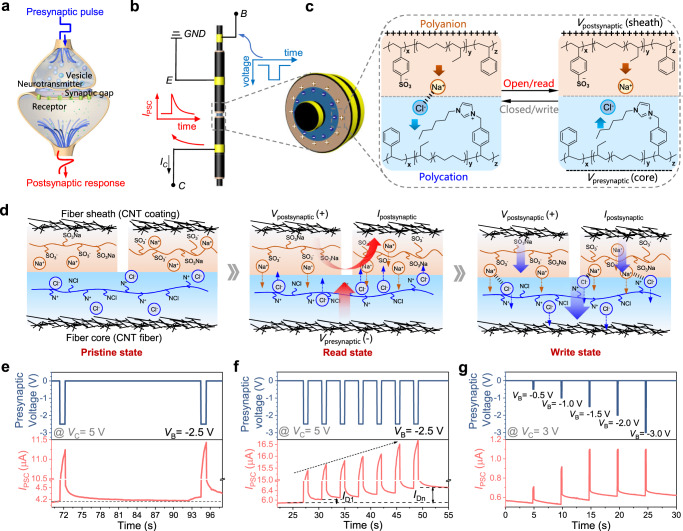
Synaptic characteristics and switching mechanism of the fiber-shaped ionic transistor. **a** Schematic illustration of a natural biological synapse. For the function of the synapse, the advent of an action potential releases neurotransmitters that aid ion channels for signal transmission from the presynaptic pulse to the postsynaptic response. **b** Schematic illustration of the three-terminal synaptic IBJT. The emitter and collector are used as the postsynaptic output terminals, while a presynaptic voltage applied on the base will trigger a postsynaptic current (*I*_PSC_) in the ionic-junction fiber. The right schematic presents the cross-section of the synaptic IBJT device. **c** A negative presynaptic potential (*V*_presynaptic_) drove Cl^−^ into the postsynaptic electrode while Na^+^ accumulated near the heterojunction in the device due to the application of the postsynaptic voltage (*V*_postsynaptic_) during an open-read operation. **d** Schematic explaining the decoupling of the read and write operations of synaptic IBJT. **e** Paired pulse facilitation (PPF). Two equally sized 500 ms pulses are applied shortly after each other to facilitate an increase in the current of the second pulse depending on the time delay between the two pulses. The two pulses did not permanently change the conductivity of the device as the current reaches the initial baseline after 23 s. **f** When multiple presynaptic voltages are applied, the PPF (*I*_D_) can be manifested by the ratio between the *I*_PSC_ measured immediately after the first pulse current (*I*_D1_) and the last pulse current (*I*_Dn_). **g** *I*_PSC_ is triggered by multiple presynaptic pulses with a consistent duration time (180 ms) and different amplitudes (−0.5, −1.0, −1.5, −2.0, and −3.0 V). Source data are provided as a Source Data file.

Neuron-like functions of the fiber-shaped ionic-junction logic device were explored to match the demand of neural signal transmission. Ion-based memristor is an electronic device with hysteretic conductance, which can serve as an elementary component for ion-based neuromorphic systems (Fig. 4a). In synaptic transistors recently reported, ions behave like neurotransmitters, sending signals between the ends to form artificial synapses^41^. The transistor can remember previous activity by retaining stored data from trapped ions, allowing it to develop long-term plasticity (LTP)^42^. Figure 4b presented a schematic illustration of the synaptic performance test of our fiber-shaped ionic synaptic transistors device. For the fiber-shaped device architecture, the CNT fiber acted as the axon of a presynaptic. It transferred the presynaptic pulse to the post neuron to convert onto the corresponding postsynaptic current (*I*_PSC_) via Na^+^ and Cl^−^ transport, as shown in Fig. 4c. For ionic synaptic transistors, which are more like a series connection of two two-terminal ionic memristors (fiber-shaped ionic diodes), a similar memcapacitor can be created by a series capacitor^43^.

Similar to the working mechanism of IBJT, as shown in Fig. 4d, Na^+^ accumulated on the SEBS-SN side near the heterojunction in the device due to the application of the postsynaptic voltage (*V*_postsynaptic_). Upon applying a negative presynaptic potential (*V*_presynaptic_) to the CNT fiber electrode, the flow pathways of Cl^−^ is from the presynaptic electrode into the postsynaptic electrode through the electrolyte, and oppositely charged ions aggregating at the heterojunction interface to form an efficient ionic current, resulting in an effective *I*_PSC_. While empowering current continuity induced by ion transport, the electrolyte also serves as a barrier to electron transport, holding the electrode conductance state after the applied presynaptic potential^44^. When *V*_presynaptic_ is removed, interestingly, Cl^−^ cannot immediately migrate back to the original position due to the electrostatic attraction of oppositely charged ions at the interface of the heterojunction, which leads to maintaining a certain ionic current and memcapacitor, that is, a memristive effect is produced^43^. The phenomenon induced by the accumulation of ions in the channel, is analogical to the short-term synaptic facilitation (ms to s), occurring in biological synapses encountering repeated action potentials resulting from the accumulation of excess Ca^2+^ and neurotransmitters. Fig. 4e–g verified the neuromorphic functionality of fiber-shaped ionic synaptic transistors by demonstrating signal processing similar to biological paired-pulse facilitation (PPF) and short-term plasticity (STP). PPF is requisite for decoding biological temporal information, which depicts the phenomenon with the appearance of two consecutive presynaptic pulses. The amplitude of *I*_PSC_ induced by the second pulse is more profound than that of the first pulse, which is determined by the time interval between the two pulses.

As shown in Fig. 4e, the *I*_PSC_ is generated by a pair of presynaptic pulses (−2.5 V, 500 ms) with a pulse interval (Δ*t*) of 23 s. The amplitude of the *I*_PSC_ generated by the second presynaptic pulse is obviously larger than that of the first one, evidencing the non-permanent change in synaptic weight resulting from the application of a single pulse pair. When multiple presynaptic voltages are applied, the PPF (*I*_D_) can be manifested by the ratio between the *I*_PSC_ measured immediately after the first pulse (*I*_D1_) and the last pulse (*I*_Dn_), as shown in Fig. 4f. Multiple presynaptic pulses with the same duration time (180 ms) and various amplitudes (−0.5, −1.0, −1.5, −2.0, and −3.0 V) were applied to the presynaptic neuron electrode, and a postsynaptic voltage (*V*_postsynaptic_ = 3.0 V) was applied for the *I*_PSC_ measurement (Fig. 4g). The *I*_PSC_ exhibited a sharp increase after applying the presynaptic pulse and gradually decayed to the resting current (0.5 μA). The value of the *I*_PSC_ peak increased systematically with amplitudes of presynaptic pulses, which is analogical to the behavior of biological excitatory synapse^45^. This experiment confirmed the synaptic characteristics of ionic-junction fibers and inspired applications for realizing the learning and memory function similar to neurons in living organisms in the future.

### Application of nerve signal transmission of the ionic-junction logic fiber in vivo

Peripheral nerve injuries (PNI), which cause the loss of sensation and muscle control, have been a significant clinical challenge for decades. End-to-end anastomosis without tension is still the gold standard in microsurgical nerve repair. However, the difficulties of nerve anastomosis will be exacerbated when the proximal stump of the injured nerve is surgically inaccessible to the distal nerve stump of the targeted muscle^46^. A nerve graft will be a must in this condition if the traditional end-to-end anastomosis is used. However, an end-to-side anastomosis that coaptates the distal stump of the injured nerve to the lateral side of an adjacent uninjured nerve can be an effective alternative. Besides, as new surgical strategies such as contralateral C7 nerve root transfer via pre-spinal or retro-spinal route and others have been reported in recent years, corresponding rehabilitation of various kinds, including electrical stimulation (ES), are in high demand in clinical practice^46,47^.

**Fig. 5:**
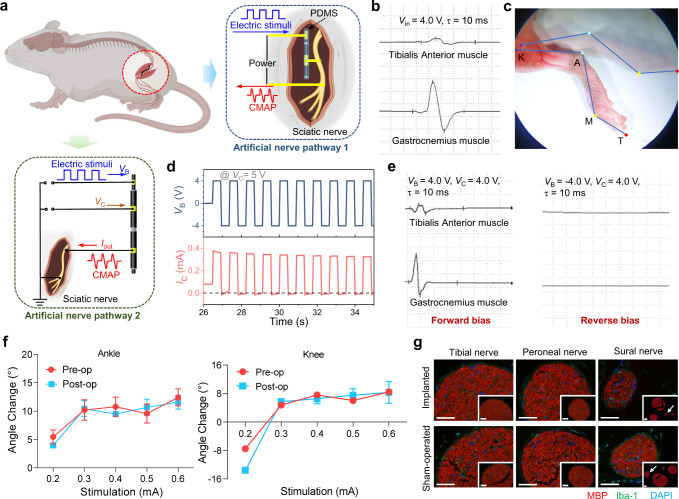
Application of nerve signal transmission for artificial nerve pathway of the ionic-junction fiber and its biocompatibility in vivo. **a** Schematic illustration of two signal transmission pathways made of a fiber-shaped ionic-junction fiber and a fiber-shaped IBJT, respectively. *I*_out_, output current (Note, the model of the mouse in the illustration created with Biorender.com.). **b** Compound muscle action potential (CMAPs) of the Gastrocnemius muscle and Tibialis Anterior muscle at a stimulating voltage of 4.0 V with a pulse width of 10 ms. *V*_in_, input voltage. **c** Angle analysis of extension and flexion in the ankle joint induced when a fiber-shaped device was implanted in contact with the sciatic nerve. The knee joint (K), ankle joint (A), metatarsophalangeal joint (M), and the end of toes (T) were labeled on the hindlimb of the mouse. **d** Output characteristic (*I*_C_-*V*_C_) curves of fiber-shaped IBJT under alternating potentials of a forward bias of +4.0 V and a reverse bias of −4.0 V. (Applied voltage is plotted on the top, while the corresponding current is demonstrated on the bottom) **e** CMAPs of the Gastrocnemius muscle and Tibialis Anterior muscle induced from IBJT when applied a square wave voltage (*V*_B_) of forward 4.0 V and reverse −4.0 V with a stimulus pulse width of 10 ms, respectively, as well as the *V*_C_ is 4.0 V. **f** Comparison of action angles (ankle joints and knee joints) by stimulating the sciatic nerve when the ionic-junction fiber was implanted in the mouse before and after 2 weeks. Pre-op, Pre-operation; Post-op, Post-operation. Data are shown as mean values ± s.e.m., *n*  =  4 biologically independent experiments. **g** Immunofluorescence staining of three main branches of the sciatic nerve after a two-week ionic-junction fiber implantation (micrographs, images representative of three experiments, scale bar = 50 μm). MBP myelin basic protein, Iba1 ionized calcium-binding adapter molecule-1; DAPI 4’,6-diamidino-2-phenylindole. Source data are provided as a Source Data file.

Based on the structural advantage of our fiber-shaped ionic diodes, we designed a connection, which simulates end-to-side anastomosis, between our ionic-junction fibers and the sciatic nerve as an additional neural pathway for electrical stimulation in vivo (Fig. 5a). The core layer of the fiber device was connected to the positive electrode of the signal source and sheath layer was connected to the upstream of the mouse sciatic nerve, while the downstream was grounded (Note, in the stimulation experiment, the position of polyanions and polycations are exchanged). Supplementary Fig. 20 exhibited an intraoperative image of real connections between the fiber device and the sciatic nerve with flexible metal electrodes. After being stimulated by a series of monophasic rectangle waves, the fine movement of the lower limb muscles can be induced and compound muscle action potential (CMAP) of tibial anterior muscle was thus recorded by the electromyogram recording needle electrodes (Fig. 5b)^48^. In addition, the induced action potentials led to the contraction of biceps femoris as well as calf and foot muscles, so the movements of the hindlimb, especially ankle and metacarpophalangeal (MP) joints can be observed. A high-speed camcorder was used to record the movements (Supplementary Movie 2). In detail, the movements of the ankle joint and MP joint were induced successively, and the angle changes of both joints were labeled in Fig. 5c. It was worth noting that higher voltages were detrimental to living organisms because some biological cells and tissues may be damaged due to the larger ionic resistance of the device^49^. To reduce the voltage, a current-to-voltage signal amplifier converter can be applied between the ionic-junction fiber and the sciatic nerve (Supplementary Fig. 21), which resulted in inducing a large movement of the hindlimb at a voltage of 0.5 V (Supplementary Movie 3)^16^. Moreover, a flexible AgNWs@PDMS electrode was also found to fit the nerve well without causing damage to the nerve (Supplementary Fig. 22, Supplementary Movie 4). However, it is difficult for fiber-shaped ionic diodes to realize the rapid cut-off of nerve signal when applied a reverse bias due to the relatively long transition time (Supplementary Fig. 15b). In order to improve, two fiber-shaped ionic diodes were integrated in series to construct a fiber-shaped IBJT, achieving a sub-second transition time as well as an asymmetric output current (Fig. 5d, Supplementary Fig. 23a). Consequently, the IBJT connected to the sciatic nerve successfully induced CMAPs when applied the forward voltage while the reverse voltage did not (Fig. 5e, Supplementary Fig. 23b, Supplementary Movie 5). The unidirectional stimulation of the nerve is potentially valuable in some clinical applications as presented in Supplementary Methods 9.

Safety and biocompatibility issues are essential for the long-term implantation of devices. When injecting electric currents into the tissue from these electron-conducting electrodes, electrochemical reactions may occur, and thus the resulted environment changes at the neural interface, such as the production of hazardous chemicals, pH change, or generation of local heat, poses serious issues (like acute inflammation and chronic neural disorder) to the tissue^50^. Therefore, the biocompatibility and mechanical properties of our ionic-junction fibers were tested. Firstly, stress-strain and bending tests illustrate good softness and cycling stability (Supplementary Figs. 24, 25). Then the 1 cm-long ionic-junction fiber was implanted and attached to the sciatic nerve and then the muscles above were sutured firmly to ensure close contact of the ionic-junction fiber and sciatic nerve. Angle analysis for hindlimb movements induced by electrical stimulation after implantation for 2 weeks can further prove that the device has no obvious long-term damage to the sciatic nerve in vivo (Fig. 5f, Supplementary Fig. 26, Supplementary Movie 6). Immunofluorescence staining of three sciatic nerve branches including the tibial nerve (TN), peroneal nerve (PN), and sural nerve (SN) indicated that there was negligible inflammation response in both implant and sham-operated side (Fig. 5g)^2^. Moreover, the mean sheath thickness (MST) and mean optical density (MOD) of myelin basic protein (MBP) were analyzed quantitatively. The histograms were shown in Supplementary Fig. 27. Since TN and PN were mixed nerves containing motor and sensory axons while SN was a pure sensory nerve, the MST of TN and PN was greater than that of SN. However, there was a negligible difference when MST of the implant and sham-operated side were compared. The results of MOD quantitative analysis were similar. Both results above indicate that the implantation of ionic-junction fiber for 2 weeks exerts little effect on the density and thickness of the myelin sheath, and the normal structure of axons and myelin sheath were maintained. The extra cell viability tests further confirmed that the encapsulation to the ionic-junction fiber can also significantly reduce the toxicity to nerve cells (Supplementary Fig. 28). Additionally, Ag-Au nanowires are synthesized and mixed with carbon nanotubes as Ag-Au nanowire @CNT electrode to further improve the biological stability of devices without reducing their performance (Supplementary Figs. 29, 30)^51^.

In conclusion, our experiments realized the unidirectional ES signal transmission function of ionic-junction fiber in vivo and confirmed its biocompatibility. The fine movements of hindlimb joints can be induced via our device while nerve immunofluorescence staining indicated that there was no significant inflammation response and changes of peripheral nerve normal structure after the 2-week implantation, and the ionic-junction fiber maintained its appearance and mechanical features as well. Therefore, both the safety and functional stability of our device gives inspiration for future clinical applications. More excitingly, because the CNT coating layer has a relatively large area, more than one downstream nerve can be coaptated to the ionic-junction fiber sheath through the end-to-side method. As patients with arm paralysis generally have variable motor dysfunction, with subtle adjustments made to the structure of our device, more precise and personalized therapeutic effects based on targeted highly selective multiple nerve stimulations could be realized in light of individualized rehabilitation requirements.

## Discussion

In summary, the concept of the IOCG fabrication technique was proposed to develop a polyelectrolyte heterojunction-based electron-ion mixed logic fiber, which can carry out unceasing preparation at a kilometer scale. The fiber-shaped device demonstrated primary ionic diodes and IBJT functions, implementing a “0-1” rectification and “on-off” switch of input signals with ions as charge carriers inside the device. The rectification ratio of the prepared fiber-shaped ionic diode reaches 30, and the switching ratio of the fiber-shaped ionic transistor is as high as 50. Based on that, two types of ionic-junction logic gates were constructed to realize AND and OR Boolean operations, which paved the way for designing ionic digital logic and ionic computing fiber devices. Moreover, an obvious synaptic function was observed in this ionic transistor due to the presence of memory capacitance.

The readily prepared ionic-junction fibers were end-to-side-anastomosed with the biological nerve to validate their potential in PNI rehabilitations. Excellent geometric matching and stable contact interfaces with biological nerves were observed. Our experiments confirmed that the ionic-junction fiber can transmit electrical stimulation signals in vivo while having a negligible side effect on biological nerves, which provided direction for fiber devices used for clinical applications such as nerve repair and rehabilitation in the future. All the results indicated that ionic-junction fibers have plenty of room in developing biomedical devices for diagnostics and therapeutics, biomimetic neuron computer interfaces, and brain-like intelligence. As well, the concept of IOCG could be employed as a facile way for scalable production of ionic-junction fibers and provide a valuable methodology for practical low-cost fabrication of organic ionic logical devices.

## Methods

### Materials preparation

The commercially available triblock copolymer of poly(styrene-b-(ethylene-co-butylene)-b-styrene) (SEBS, *M*_n_ = 330,000, KRATON in America) with 31 wt% of styrene structural units were used as received. Carbon nanotubes (CNT, TNF400) fibers were obtained from Timesnano (Sichuan, China). Thin multiwall carbon nanotubes (MWCNTs, 90%, NC7000) were purchased from Nanocyl S. A. Rue de l’ Essor (4 B-5060 Sambreville, Belgium). Chlorotrimethylsilane (≥97.0%), paraformaldehyde (95%), anhydrous tin tetrachloride (SnCl_4_, 98%), ethyl cellulose (AR), and 1-hexylimidazole (99%) were purchased from Sigma-Aldrich. Additionally, unless otherwise noted, commercially available reagents such as sulfuric acid (95-98%), acetic anhydride (98.5%), and sodium chloride (AR) were used without further purification.

#### Coating of polyelectrolyte precursor

CSEBS slurry, 1-hexylimidazole, deionized water, SSEBS slurry, and NaCl solution were sequentially placed in the coating tank to carry out a series of process steps including CSEBS coating, quaternization reaction, small molecule removal, SSEBS coating, and ion exchange (Supplementary Fig. 6).

##### Coating of CSEBS and following in-situ quaternization for the synthesis of SEBS-IM (polycation) on the fiber

For the coating of polycation slurry, CNT fiber (diameter 60-80 μm) was firstly immersed in the previously prepared CSEBS slurry and continuously pulled out via an electric machine, which resulting product was named CNT@CSEBS fiber. The coating layer was dried by passing through two 120 °C furnaces after coating. Next, the resulting CNT@CSEBS fiber was further immersed in 1-hexylimidazole solution at 80 °C for the in-situ heterogeneous quaternization to prepare SEBS-IM polycation via an easy Menshutkin reaction^52^. Following process steps containing washing in deionized water and drying at 120 °C were performed for the removal of reactants, which resulting product was named CNT@SEBS-IM fiber (Supplementary Fig. 7a).

##### Coating of SSEBS and following in-situ ion exchange for the synthesis of SEBS-SN (polyanion) on the fiber

Before coating the polyanion slurry, part of the area of previously prepared CNT@SEBS-IM fiber was covered by a masking tape (VHB 4905, 3M) to disconnect the polyanion and CNT coating layer, forming the channel of IBJTs. Then fibers were immersed in the SSEBS slurry and continuously drawn out by a starching machine, which resulting product was named CNT@SEBS-IM@SSEBS fiber. The coating layer was dried after passing through two 120 °C furnaces and then was further immersed in NaCl solution at 80 °C for the in-situ ion exchange to yield the SEBS-SN polyanion with sodium ions. Following process steps containing washing in deionized water and drying at 120 °C were performed to remove small molecules, the resulting product was named CNT@SEBS-IM@SEBS-SN fiber (Supplementary Fig. 7b).

The SEBS-IM is nearly hydrophobic due to the grafting of hexylimidazolium with a long alkyl chain, while SEBS-SN is hygroscopic; it can absorb up to ~30 wt% of water in ambient conditions. Therefore, we stored SEBS-SN in a desiccator with a drying agent and heated it to 60 °C under a vacuum for 3 h before use. After drying, a small amount of residual water (<2 wt%) was observed in SEBS-SN, as evidenced by ATR-FTIR (Supplementary Fig. 4a) and TGA (Supplementary Fig. 4b). CA tests can also prove this phenomenon (Supplementary Fig. 5).

### Spray coating of carbon nanotube slurries

To prepare CNT@SEBS-IM@SEBS-SN fiber devices, we used 5 ml of carbon nanotube solution for spray coating, resulting in a weight increase of 1.0 ± 0.3 mg. We named the product CNT@SEBS-IM@SEBS-SN@CNT fiber. An SEM (JSM-7500F) and optical images of the deposited carbon nanotube are shown in Supplementary Figs. 7c, 8a, respectively.

### Preparation of SEBS-based ionic-junction fiber devices

We use copper tape coated with silver paste as the electrode current collector for device testing, and the fiber-shaped ionic diode was designed: conductive layers (copper tape with silver paste, the width is 0.5 cm) formed an electron collector layer, which was tightly attached to the surfaces of the core (CNT fiber) and sheath (CNT coating) for the electrical output measurements, respectively (The pads distance between the core layer and sheath layer is 3 cm). In summary, we successfully fabricated ionic-junction fiber diode devices (Supplementary Fig. 10).

For fiber-shaped ionic transistors, two outer SEBS-SN layers separated by a lateral gap of <1 mm were attached to a medial SEBS-IM film. Similarly, we also used copper tape coated with silver paste as the electrode current collector and the core layer of the fiber were used as the base of the BJT. In contrast, the two separated sheath layers were used as the emitter and collector of the transistor, respectively. The structure can also be used as a test device for synapse performance (Supplementary Fig. 18). Finally, all devices were encapsulated between two layers of VHB 4905 adhesive to insulate the ionic-junction fiber devices electrically.

### Electrochemical analysis

The *I*-*V* characteristics of the device were measured with a series of sweep scan rates (1 mV/s-1 V/s) by using a computer-controlled electrochemical workstation (Princeton, PMC CHS08A) at 25 °C. Unless stated otherwise, the positive terminal (or working electrode) was connected to the CNT coating layer (sheath) and the negative terminal (or counter and reference electrodes) to the CNT fiber (core) for electrochemical analysis. Electrochemical Impedance Spectroscopy (EIS) was measured over a frequency range of 1.0 MHz-0.1 Hz. The electrochemical workstation measured the current density of the fiber-shaped ionic-junction logic device as a function of time under various direct current (DC) biases.

#### Demonstration of ionic-junction logic circuits

Two ionic-junction logic gates were also constructed: “OR” gate and “AND” gate. For electrical characteristics of ionic-junction logic circuits, the electrical outputs were recorded by an oscilloscope (Tektronix, MSO44), and the electrical inputs to the fiber-shaped ionic-junction logic circuits were applied using a function generator (Tektronix, AFG31000). For the “AND” gate, we use a DC voltage source (Dr. Meter HY1803D) as its power supply (*V*_cc_ = 5 V) through two resistors connected in series: *R*_1_ = 10 MΩ and *R*_2_ = 100 MΩ. The two input voltage signals *V*_A_ and *V*_B_ were applied on the two CNT fibers of the gate respectively, and the voltage on the CNT coating (*V*_out_) served as the output signal of the gate^38^. Similarly, an ionic “OR” gate was integrated based on a converse connection of two fiber-shaped ionic diodes.

#### IBJT and synaptic characterization of the ionic-junction logic fiber

Electrical characteristics of the fiber-shaped transistor and the synaptic device were measured using a Keithley 4200 semiconductor parameter analyzer system.

### Animal experiments of the ionic-junction fiber in vivo

Relevant details about animal experiments are available within the Supplementary Information files.

### Theoretic modeling

Detailed procedures for theoretic modeling of potential distribution profiles in the ionic junction are available within the Supplementary Information files.

### Reporting summary

Further information on research design is available in the Nature Portfolio Reporting Summary linked to this article.

## Supplementary information

Supplementary Information

Description of Additional Supplementary Files

Supplementary Movie 1

Supplementary Movie 2

Supplementary Movie 3

Supplementary Movie 4

Supplementary Movie 5

Supplementary Movie 6

Reporting Summary

## Data Availability

All data supporting the findings of this study are available within this article and Supplementary Information. The data generated in this study are provided in the Source Data file. Source data are provided with this paper.

## Source data

Source Data
