# Supplementary material for: Integrated opposite charge grafting induced ionic-junction fiber
Source: Nat Commun. 2023 Apr 24;14:2355. doi: 10.1038/s41467-023-37884-0 (PMC10126126; doi:10.1038/s41467-023-37884-0)
Supplement: Supplementary file 1 — Supplementary Information [file 41467_2023_37884_MOESM1_ESM.pdf]

Supporting Information

Integrated Opposite Charge Grafting Induced Ionic-junction Fiber

Yi Xing[1]†, Mingjie Zhou[2]†, Yueguang Si[3], Chi-Yuan Yang[4], Liang-Wen Feng[5], Qilin Wu[1], Fei Wang[2], Xiaomin Wang[2], Wei Huang[6], Yuhua Cheng[6], Ruilin Zhang[7], Xiaozheng Duan[7], Jun Liu[8], Ping Song[8], Hengda Sun[1], Hongzhi Wang[1], Jiayi Zhang[3], Su Jiang[2], Meifang Zhu[1], Gang Wang[1]*

[1] State Key Laboratory for Modification of Chemical Fibers and Polymer Materials, College of Materials Science and Engineering, Donghua University, Shanghai 201620, China

[2] Department of Hand Surgery, Center for the Reconstruction of Limb Function, National Clinical Research Center for Aging and Medicine, Huashan Hospital; Department of Hand and Upper Extremity Surgery, Jing'an District Central Hospital; NHC Key Laboratory of Hand Reconstruction, Shanghai Key Laboratory of Peripheral Nerve and Microsurgery, Institute of Hand Surgery, Fudan University, Shanghai, 200040, China

[3] State Key Laboratory of Medical Neurobiology, MOE Frontiers Center for Brain Science, Institute of Brain Science, Department of Ophthalmology, Zhongshan Hospital, Fudan University, Shanghai 200032, China.

[4] Laboratory of Organic Electronics, Department of Science and Technology, Linköping University, SE-601 74, Norrköping, Sweden

[5] College of Chemistry, Sichuan University, Chengdu, 610064, China

[6] School of Automation Engineering, University of Electronic Science and Technology of China, Chengdu, 611731, China

[7] State Key Laboratory of Polymer Physics and Chemistry, Changchun Institute of Applied Chemistry, Chinese Academy of Sciences, Changchun, Jilin 130022, China

[8] National Key Laboratory on Electromagnetic Environmental Effects and Eletro-optical Engineering, Nanjing, 210007, China

*Corresponding author. Email: gwf8707@dhu.edu.cn (G.W.)

†These authors contributed equally to this work.

This file includes

Supplementary Methods 1. Preparation of polyelectrolyte precursor.

Supplementary Methods 2. Preparation of carbon nanotube slurries.

Supplementary Methods 3. Fabrication of AgNWs@CNTs electrodes.

Supplementary Methods 4. Process analysis and optimization.

Supplementary Methods 5. Cyclic bending testing of the ionic-junction fiber.

Supplementary Methods 6. Characterization of ionic-junction fiber.

Supplementary Methods 7. Animals.

Supplementary Methods 8. Electrical stimulation (ES) signal transmission on the sciatic nerve *via* the ionic-junction fiber.

Supplementary Methods 9. Application senario of unidirectional stimulation to the nerve induced by fiber-shaped ionic bipolar junction transistors (IBJTs).

Supplementary Methods 10. Fabrication of the flexible electrode connecting the fiber device.

Supplementary Methods 11. Ionic-junction fiber implantation and immunofluorescence staining**.**

Supplementary Methods 12. Hindlimb movement analysis.

Supplementary Methods 13. Assessment of locomotor activity during Gait analysis, ladder rung walking test and motion analysis.

Supplementary Methods 14. Cytotoxicity and biocompatibility of ionic-junction fiber.

Supplementary Methods 15. Statistical analysis.

Supplementary Methods 16. Theoretic modeling of potential distribution profile in the ionic-junction fiber.

Supplementary Fig. 1. The synthesis process and nuclear magnetic resonance (NMR) spectra of SEBS-IM-x copolymer with different degrees of chloromethylation.

Supplementary Fig. 2. The synthesis process and attenuated total reflection Flourier transformed infrared (ATR-FTIR) spectra of SEBS-SN copolymer with different degrees of sulfonation.

Supplementary Fig. 3. Differential scanning calorimetry (DSC) thermograms of SEBS-IM and SEBS-SN polyelectrolytes.

Supplementary Fig. 4. Contents of residual water for SEBS-IM and SEBS-SN polyelectrolytes.

Supplementary Fig. 5. Contact angle (CA) of dried polyelectrolyte membranes.

Supplementary Fig. 6. Schematic diagram of the home-made integrated fabrication instrument for ionic-junction fiber.

Supplementary Fig. 7. Optical photograph of the completed fiber.

Supplementary Fig. 8. SEM images of the CNT fiber before and after surface coating.

Supplementary Fig. 9. Simulated induced potential distribution profile along the thickness direction of the fiber-shaped ionic diode by at zero bias.

Supplementary Fig. 10. Schematic illustration of the preparation of the fiber-shaped ionic diode.

Supplementary Fig. 11. Nyquist plot of SEBS-IM / SEBS-SN under +3.0 V.

Supplementary Fig. 12. CV curves of ionic-junction fibers at different voltage ranges with a scan rate of 10 mV/s.

Supplementary Fig. 13. Full *J-V* characteristics of ionic polyelectrolyte junctions (SEBS-IM/SEBS-IM, SEBS-SN/SEBS-SN, and SEBS-IM/SEBS-SN).

Supplementary Fig. 14. Comparison of the rectification performance of reported solid-state ionic diode devices with our fiber-shape d ionic diodes.

Supplementary Fig. 15. Transient response characteristics of the fiber-shaped ionic diode (Φ = 153 μm, Φ, diameter).

Supplementary Fig. 16. Rectification ratio of the fiber-shaped ionic diode with two diameters at different scanning rates from 100 mV s^{-1} to 1 mV s^{-1}.

Supplementary Fig. 17. The rectifying behaviors at different scan rates of 0.5 mV/s were evaluated via linear sweep voltammetry from -3 V ~ 3 V and -6 V ~ 6 V, respectively.

Supplementary Fig. 18. Schematic illustration of the preparation of the fiber-shaped IBJT.

Supplementary Fig. 19. Demonstration of ionic-junction logic gates based on multiple fiber-shaped ionic diodes.

Supplementary Fig. 20. An intraoperative image of fibers effectively connect with the nerve through flexible metal electrodes in a mouse (Scale bar: 3 mm).

Supplementary Fig. 21. A current-to-voltage signal amplifier converter was applied between the ionic-junction fiber and the sciatic nerve.

Supplementary Fig. 22. Difference of CMAPs induced by a flexible electrode connecting the fiber device.

Supplementary Fig. 23. Output characteristic of signal transmission pathways made of a fiber-shaped IBJT.

Supplementary Fig. 24. Mechanical properties of the component of the polyelectrolytes in the fiber.

Supplementary Fig. 25. Comparison of rectification retention performance of fibers with CNT electrodes and with CNT@AgNWs electrodes under cyclic bending.

Supplementary Fig. 26. Assessment of locomotor activity during Gait analysis, ladder rung walking test and motion analysis.

Supplementary Fig. 27. Quantitative analysis of mean sheath thickness (MST) and mean optical density (MOD) of sciatic nerve.

Supplementary Fig. 28. Cytotoxicity of ionic-junction fiber.

Supplementary Fig. 29. Characterization of the Ag-Au nanowire.

Supplementary Fig. 30. Output signals of two artificial nerve pathway that constructed with Ag-Au nanowires.

Supplementary Table 1. Fitting parameters for circuit components in the equivalent circuit model under a forward bias of +3.0 V.

Supplementary References 1-26

Supplementary Methods

Preparation and Characterization

1. Preparation of polyelectrolyte precursor. As shown in Supplementary Fig. 1a, Chloromethylated SEBS (CSEBS-x) with different degrees of functionalization was synthesized by a general method[1]. Typically, SEBS (4.0 g, 19.3 mmol), p-formaldehyde (5.8 g, 193.0 mmol), and Chlorotrimethylsilane (24.4 ml, 193.0 mmol) were dissolved in 110 ml $CHCl_3$ with a magnetic stirrer under N_2 atmosphere. Then anhydrous tin tetrachloride (0.5 ml, 3.9 mmol) in $CHCl_3$ (50 ml) was added at 45°C. The mixture was reacted at 50°C under an N_2 for 72h. The white flocculent product was obtained in methanol. Finally, the CSEBS-100 copolymer was dried under vacuum at 60°C for 24 h[2]. The degree of chloromethylation (x values) in CSEBS-x copolymers was calculated based on ^{1}H Nuclear Magnetic Resonance (NMR) spectra, which can be adjusted by controling the reaction time (see Supplementary Fig. 1b, c).

Sulfonation of SEBS (SSEBS) and determination of sulfonation degree: As shown in Supplementary Fig. 2a, acetyl sulfate was obtained by mixing a certain amount of sulfuric acid with acetic anhydride in $CHCl_3$ at 0 ~ 5°C. The theoretical sulfonation degree was from 10% to 40%. 5 g of SEBS was dissolved in 50 ml of $CHCl_3$ at 25°C and then the acetyl sulfate was added to the transparent SEBS solution. The solution was stirred for 24 h. Finally, 10 ml of isopropyl alcohol was added to terminate the reaction. SSEBS was obtained and washed with deionized water until the pH of the solution was neutral.

To test the sulfonation degree of SSEBS, the confirmation of sulfonation was performed using a fourier transform infrared spectrometer (FTIR, Spectrum BXII, Perkin Elmer) in a full range (see Supplementary Fig. 2b). The sulfonation degree of SSEBS was calculated by the sulfur weight percent obtained from the result of Elemental analysis (EA, CHNS-932, Leco) measurement (see Supplementary Fig. 2c)[3]. The equation can be expressed as follows:

$$\textit{Sulfonation degree} = \frac{moles\ of\ sulfonated\ styrene}{moles\ of\ sulfonated\ SEBS} \times 100\% \quad (1)$$

Preparation of polyelectrolyte precursor slurries: The slurry was prepared by using a mixer. For the CSEBS slurry, CSEBS copolymers having different grafting degrees were dissolved in a mixture of chloroform and toluene with a proportion of a 7 to 3 ratio in a planetary centrifugal mixer and stirred for 2 h. The solid content of the slurry is a key parameter affecting the thickness of ionic polyelectrolyte layers. If the solid content is too low, the slurry viscosity is insufficient, and the polyelectrolyte cannot form a uniform layer on the fiber. The slurry viscosity could be enhanced by increasing the solid content of active material in the slurry.

However, excessive CSEBS rubber would form polymer bulges or even polymer filaments on the fibers, resulting in more non-uniform devices, which directly affects the performance of the device[4]. After careful optimization, the solid content of CSEBS was 10 wt%. For the SSEBS slurry, in the same way, copolymers were dissolved in a mixture of tetrahydrofuran (THF) and toluene with a proportion of a 7 to 3 ratio with a solid content of 10 wt%. The two mixed solutions are poured into the coating tank to prepare the following coating preparation.

2. Preparation of carbon nanotube slurries. Ethylcellulose was dissolved in ethanol at a 2 mg ml^{-1}. 200 mg MWCNT powder was added to the above solution at a 2 mg ml^{-1}. The solution (40 ml) was mixed with magnetic stirring in a high shear mixer (BILON92-II, Shanghai Bilang) for 30 min at a power of 150 W in an ice water bath to obtain a well-dispersed CNT slurry[5]. Undispersed carbon nanotubes were separated from the solution by centrifugation for 10 min at 2500 g.

3. Fabrication of AgNWs@CNTs electrodes. High conductive AgNWs (average diameter is 40 ~ 60 nm and length is 15 ~ 25 μm) were mixed with CNTs by ultrasonic stirring to form a conductive interconnection network, which was used to improve the mechanical durability of the surface electrode during the fiber implantation.

4. Process analysis and optimization. To obtain fibers with better device performance and precisely control the rectification ratio of ion diodes, it is important to accurately control the thicknesses of polyelectrolyte layers to achieve a high loading weight. According to the Landau-Levich law, the thickness of the polyelectrolyte layer largely depends on the balance between viscous[6]. The loading weight of the polyelectrolyte can be precisely adjusted by changing the slurry viscosity and coating speed. After optimization, the diameter of the fiber device is 500 ± 50 μm, and weight density per meter of fiber is maintained with increasing electrode length (60 ± 5 mg m^{-1}).

5. Cyclic bending testing of the ionic-junction fiber. we proceed with cyclic bending testing of the ionic-junction fiber by using the home-made equipment for stretching of flexible electronic devices and specific steps are as follows: The PET substrate with the ionic-junction fiber on it was placed on two X-Y-Z mechanical stages with a moving step of 2 mm, and the ends of the substrates were secured to each side of the workbench with adhesive tape. The bending angle of the fiber is controlled by adjusting the distance of the workbench.

6. Characterization of ionic-junction fiber. ^{1}H-NMR spectra were tested on a Bruker DPX-400 spectrometer using $CDCl_3$ as solvent. The ATR-FTIR of polymer films was recorded from 4000 to 400 cm^{-1} with a 4 cm^{-1} resolution in 64 scans on a PE-1710 spectrometer. A Contact Angle Goniometer (JC2000D1, Powereach®) was used to determine the CA testing of dried SEBS-based polyelectrolyte film. Thermograms of SEBS-IM and SEBS-SN were measured by differential scanning calorimetry (DSC, Q200, TA instruments). SEBS-IM and SEBS-SN (~5 mg) in standard aluminum DSC pans were heated from -60 °C to 120 °C at a scan rate of 10 °C min^{-1}. The second heating cycle is shown in Supplementary Fig. 3. TGA of SEBS-IM and SEBS-SN were conducted using Q500 (TA instruments). Two polyelectrolyte samples (~15 mg) were heated to 700 °C at a rate of 10 °C min^{-1} under N_2 conditions. Mechanical properties of integrated fibers and the component of the polyelectrolytes were measured on an Instron-5969 (Instron Co., USA) mechanical testing instrument at a cross head speed of 100 mm/min. The bending properties of the ionic-junction fibers were measured on a microcomputer controlled electronic universal testing machine (C42.503Y MTS) at a cross head speed of 5 mm/min.

Animal experiments of the ionic-junction fiber in *vivo*

7. Animals. Animal care and experiments were performed in accordance with the National Institutes of Health Guide for the Care and Use of Laboratory Animals and were approved by the Animal Care and Use Committee of Shanghai Medical College of Fudan University. C57BL/6 mice were obtained from the Shanghai Laboratory Animal Center, CAS (Shanghai, China). All animals were housed at 22 °C, with 12-h light/dark cycles. Room humidity was controlled at 50%. All experiments were conducted during the light cycle.

8. Electrical stimulation (ES) signal transmission on the sciatic nerve *via* the ionic-junction fiber. C57BL/6 mice were anesthetized with 0.8-1.0% isoflurane. After shaving and skin disinfection were performed, the vastus lateralis muscle and biceps femoris muscle were bluntly dissected to expose the sciatic nerve. For electrical stimulation of the sciatic nerve, two electrical stimulation needles were positioned at the proximal and distal sides of the sciatic nerve distal stump. An electrical stimulatory pulse (duration: 0.1-10 msec, frequency: 1Hz) was delivered by the Medtronic KeyPoint Portable EMG device. And two-needle recording electrodes were inserted respectively into the tendon and the middle of the anterior tibial muscle to record CMAP. Data were collected using an AM1800 amplifier (Datawave Technology, Inc., USA) and AXON DIGIDATA 1440 A digital-analog converter (AXON Inc., BMU). The

diameter of the ionic-junction fiber is 150 ± 10 μm. Different voltages were randomly applied, and the procedure was repeated ten times.

9. Application senario of unidirectional stimulation to the nerve induced by fiber-shaped ionic bipolar junction transistors (IBJTs). The function of unidirectional conductivity can be of great importance in some application conditions. For example, the vagus nerve is composed of somatic and visceral afferent fibers (inward conducting nerve fibers that transmit impulses to the brain) and efferent fibers (outward conducting nerve fibers that transmit impulses to effectors to regulate activities such as muscle contraction or gland secretion)[7]. Upon applying a stimulus, however, action potentials are evoked bidirectionally on afferent and efferent fibers, which may generate unwanted effector responses. Such pulses may result in excessive hydrochloric acid production by stimulating the gastric glands or affect the heartbeat of patients[8]. Using the unidirectional conductivity in the device, it is expected to achieve unidirectional stimulation to the nerve to restore the motor function without causing adverse sensory reactions of patient. To verify the unidirectional conductivity of the IBJT for neural stimulation, the emitter of the IBJT was connected to the sciatic nerve, as well as the base and the collector were connected to the pulse signal generator and external power supply, respectively.

10. Fabrication of the flexible electrode connecting the fiber device. The flexible electrode is prepared as follows: 10 mg AgNWs powder (XFNANO Materials Tech Co.,Ltd, Nanjing, China) was added to the ethanol solution at a 1 mg ml^{-1}, and the solution was mixed with magnetic stirring a high shear mixer (BILON92-II, Shanghai Bilang) for 30 min at a power of 150 W to obtain a well-dispersed AgNWs slurry. Meanwhile, only the shape of the two wires was exposed by patterning the pre-cleaned glass sheet using tape. The as-prepared AgNWs solution was sprayed on the patterned glass using a commercial air spray gun (U-star, Taiwan) at a distance of ~15 cm and pressure of ~25 psi. Then the tape was removed and the prepared PDMS solution was cast on the glass containing AgNWs, placing it in an oven at 60 °C for 2h. Finally, PDMS membrane (100 μm) was peeled off and AgNWs was remained intact on PDMS membrane due to strong interfacial bonding force between the AgNWs and PDMS.

11. Ionic-junction fiber implantation and immunofluorescence staining. The ionic-junction fiber was implanted and attached to the sciatic nerve of one side, while the sciatic nerve of the other side was sham-operated. In detail, the sciatic nerve was exposed. Afterward, a 1cm-long ionic-junction fiber device was implanted, then the muscles above were sutured firmly to ensure

the close contact of the ionic-junction fiber and sciatic nerve. For the sham-operated side, the surgical procedure was repeated except that an ionic-junction fiber was not implanted. After the operation, the mouse was put in a thermotank and returned to the cage upon its full awake.

After two weeks of implant, the sciatic nerve was exposed again *via* the original surgical approach, then the sciatic nerve from the nerve root to the popliteal fossa was transected and sectioned for cross-section slice. The immunofluorescence staining of the portion near the popliteal fossa where the branches can be observed was shown as a representative in Fig. 5g.

As for immunofluorescence staining, the slices (cross-sections; 4 μm thick) were washed with 0.01M PBS (pH=7.4) 3 times (5 min each) and permeabilized in 0.5% Triton-X-100 for 30 min. The slices were then blocked in 3% BSA for 30 min at room temperature and incubated with primary antibodies overnight at 4 °C (Anti-iba1 Antibody, (Mouse, Sigma-Aldrich, (MABN92), 1:200), BMP (Rat, Novus Biologicals, (NB600-717), 1:200)). The slices were rewashed with 0.01M PBS 3 times (5 min each). Secondary antibodies (Servicebio, Inc., China) were applied to the slices and incubated at room temperature for approximately 2 h in the dark. The slices were washed three times (5 min each), stained in a DAPI solution working solution for 10 min and washed 3 more times. Finally, the slides were mounted and photographed with a confocal microscope. Then the quantitative analysis of mean sheath thickness and mean optical density of MBP for sciatic nerves from both sides was performed in ImageJ. Nikon Eclipse C1 Confocal Microscopy was used for the photographs.

12. Hindlimb movement analysis. The video of hindlimb movement was recorded using a high-speed camera and edited by Adobe PR 2020. The trajectory of the hindlimb was traced with Deeplabcut Toolbox 2.2.1, and the angle changes of each joint were exported afterwards. In detail, we define the frame before movement as the rest position and the frame with the maximal ankle and MP joint movement as the action position. The reference points were marked at the knee joint, ankle joint, MP joint, and the ends of the toes. Then the reference points were connected and joint angle data in rest and action position was measured. The change in joint angle was analyzed by comparing the rest and action positions. And MATLAB 2021a was used for drawing the reproduced hindlimb motion trajectory.

13. Assessment of locomotor activity during Gait analysis, ladder rung walking test and motion analysis. The detailed methods and results are as follows:

Gait analysis: The gait analysis can help to assess many different parameters concerning the footprints, stance and gait of mice[9]. All procedures were performed in the dark to enhance

the contrast of the paw print images. The hardware setup and the corresponding software was used to analyze the gait of unforced moving mice (see Supplementary Fig. 27a). The setup includes a hardware system with a glass corridor plate illuminated with green LED which is reflected within the glass. A high-speed video camera was underneath the illuminated glass plate to record the whole walking process. Gaitlab Version1.1 was used for quantitative assessment of animal footprints. A successful run was defined as a complete run along the corridor without any interruption or hesitation. A number of 3 replicate crossings made by the mouse was recorded. The results showed that the stride frequency, stance time and swing time was affected within 6 days post-operation, but it gradually recovered itself afterwards.

Ladder rung walking test: The ladder rung walking task can help assess motor function and measure hindlimb placing, stepping and inter-limb co-ordination[10]. The horizontal ladder rung apparatus consists of metal rungs and clear glass sidewalls. A high-speed video camera was used to record the placement of hindlimbs on the rungs during each test. Three compliant runs were chosen for data analysis. We use the 7-category rating scale from Metz GA et al. for the foot placement. The scale distinguishes between the following categories: (0)total miss (1) deep slip (2) slight slip (3) replacement (4) correction (5) partial placement, and (6) correct placement. Since the scores most frequent given were (5) partial placement and (6)correct placement, these two categories were counted as "Normal" in Supplementary Fig. 27b. No placements with scores lower than "Correction"(4) were observed in all tests from before operation to 12 days after operation. We calculate the percentage of "Normal" and "Correction" score of total steps scored for each test. The test was repeated for 3 times. The statistical results show that the motor function of mouse's hindlimb undergoing operation was affected within 6 days post-operation, because the ratio of correction (5) was relatively higher than pre-operation. However, it also returned to its preoperative level itself afterwards.

Motion analysis: The motion analysis can assess the motor function of sciatic nerve by quantitative measurement of the angle changes of mouse's knee and ankle joints[11]. The mouse were trained to run along the glass corridor. A high-speed video camera was positioned as close as possible to the mouse to achieve optimal resolution of the recorded movement. The hair of hinblimb was removed for accurate positioning of hip joint (purple), knee joint (blue), ankle joint (green), metatarsophalangeal joint(yellow) and toes(red). Deeplabcut Toolbox 2.2.1 was used for the markerless positioning of every joint. The average of maximums of the angle change of knee and ankle joints for three replicate tests was calculated for statistical analysis to reflect the motor function of hindlimb at each time point (see Supplementary Fig. 27c). The results indicate that since the device was implanted close to the knee joint, range of motion of

knee joint was obviously affected after the operation in the short term, but the mouse quickly adapted to the implanted device because it returned to its normal activities within 6 days.

14. Cytotoxicity and biocompatibility of ionic-junction fiber. PC-12 cell line were kindlprovided by Stem Cell Bank, Chinese Academy of Sciences. Specific steps are as follows: In the cell viability tests, after seeded at a density of around 5000 cells/100μL in 96 well plates, PC12 cells were precultured in pristine culture media for 12 h. Then PC12 cells are cocultured with an 5mm fiber in mixed for 5 days. Afterwards, the CCK-8 assays (Abmole Bioscience, Shanghai, China) are conducted, in which the cell viability of unencapsulated and encapsulated ionic-junction fibers of two kinds (PDMS and SEBS) was tested respectively. Moreover, live-dead cell assays are also carried out. In this work, PC12 cells after coculture with fibers for 5 days were stained with Calcein-AM/PI solution (Beyotime, Shanghai, China). Photographs for live/dead cell staining was collected by Nikon Ti2 fluorescence microscope.

15. Statistical analysis. Statistical analyses were performed in Microsoft Excel 2019 (Microsoft Corporation, Inc., USA) and GraphPad Prism 9. In Supplementary Fig. 26, the data proved to be in normal distribution using the Kolmogorov-Smirnov normality test. For the comparison of the two groups, data were analyzed using two-sided unpaired t-test. P value < 0.05 was considered statistically significant. Data are presented as the mean ± s.e.m. (standard error of the mean).

16. Theoretic modeling of potential distribution profile in the ionic-junction fiber. The potential distribution profile in Fig. 2a and Supplementary Fig. 9 was simulated with coupled Nernst-Planck-Poisson model, using a commercial finite-element software package COMSOL Multiphysics (version 5.3). In our work, there are fixed anion and mobile Na^+ present in SEBS-SN, while fixed cation and mobile Cl^- present in SEBS-IM. The mobile ions drift through the bilayer interface under applied potential φ, in that situation, the following equations are applied[12]:

$$\nabla \cdot (-\epsilon_0 \epsilon_r \nabla \varphi) = F \sum_i z_i c_i + \rho_{fix} \tag{1}$$

$$J_i = -D_i \nabla c_i - z_i u_i F c_i \nabla \varphi \tag{2}$$

$$\nabla \cdot J_i = 0 \tag{3}$$

where F is Faraday's constant; z_i is the charge number; c_i is the concentration of the mobile ions; and ρ_{fix} is the charge density of the immobilized ions in the matrix; ϵ_0 is the vacuum dielectric constant, and ϵ_r the relative permittivity assumed to be 4 for SEBS like elastomers[13]. The mobility u_i and the diffusion coefficient D_i are related by the Nernst-Einstein relation:

$$u_i = \frac{D_i}{RT} \tag{4}$$

Where R and T stand for universal gas constant, and absolute temperature. The grafting percentage used in the main text for device fabrication is 40% for SEBS-SN and 100% for SEBS-IM, respectively. Therefore, fixed charge density of 293 mol/m^3 for SEBS-SN and 596 mol/m^3 for SEBS-IM are used in the simulation, accordingly the initial mobile ion density (293 mol/m^3 for $c_{Na^+,0}$ and 596 mol/m^3 for $c_{Cl^-,0}$). Since there is sufficient volume for mobile ions to reach equilibrium with the fixed charges after crossing the heterojunction interface, and no mobile ions could penetrate further into the bulk, at and outside the target simulation boundary on x-axis direction, we assume the ion concentrations stays same with the initial value $c_{i,0}$.

Supplementary Figures and Tables

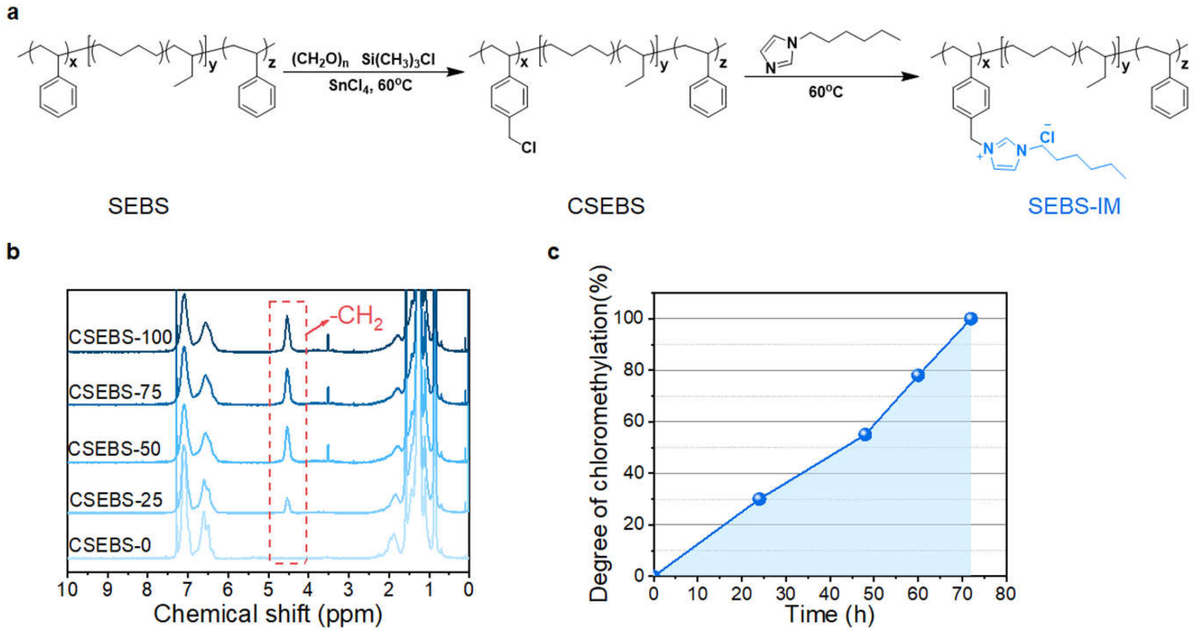

Supplementary Fig. 1. The synthesis process and nuclear magnetic resonance (NMR) spectra of SEBS-IM-x copolymer with different degrees of chloromethylation. a Synthesis scheme and **b** ^{1}H NMR spectra: ^{1}H NMR (600 MHz, $CDCl_3$). "1" is defined as the signal peak of methylene. **c** Degree of chloromethylation as a function of time. Source data are provided as a Source Data file.

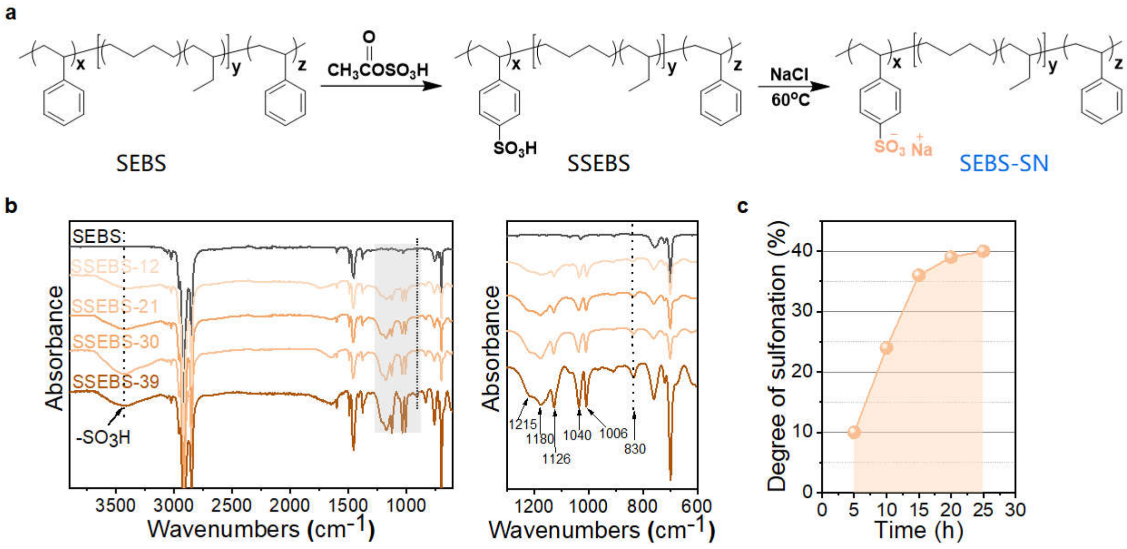

Supplementary Fig. 2. The synthesis process and attenuated total reflection Flourier transformed infrared (ATR-FTIR) spectra of SEBS-SN copolymer with different degrees of sulfonation. a Synthesis scheme and **b** ATR-FTIR spectra of sulfonation of SEBS (SSEBS) copolymer. **c** Degree of sulfonation as a function of time at 25 °C. Source data are provided as a Source Data file.

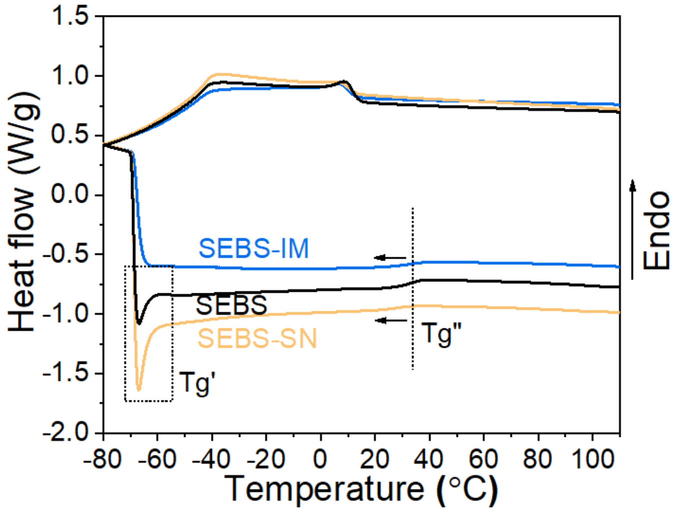

Supplementary Fig. 3. Differential scanning calorimetry (DSC) thermograms of SEBS-IM and SEBS-SN polyelectrolytes. Two glass transitions temperatures (T_g) are observed at -60 ºC (T_g') and 37 ºC (T_g") for SEBS and two grafted SEBS polyelectrolyte, with SEBS-IM showing slightly lower T_g of 35.6 ºC. Source data are provided as a Source Data file.

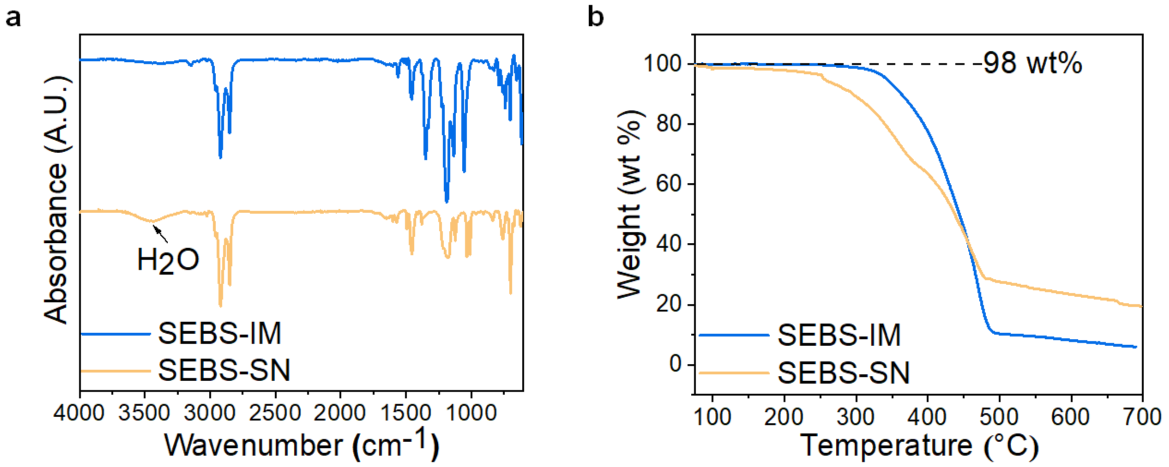

Supplementary Fig. 4. Contents of residual water for SEBS-IM and SEBS-SN polyelectrolytes. a The vibration peak of H_2O in the range of 3300 - 3500 cm^{-1} is observed for SEBS-SN in ATR-FTIR. **b** Thermogravimetric analysis (TGA) of polyelectrolytes with a heating rate of 10 ºC/min. A small amount of residual water of 2 wt% weight loss in SEBS-SN can be observed at 100 °C. Source data are provided as a Source Data file.

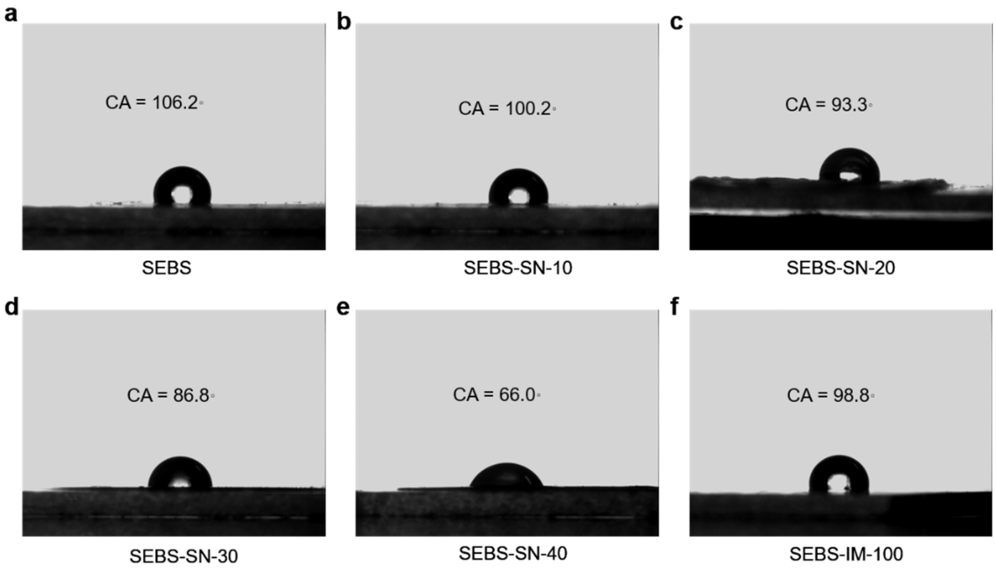

Supplementary Fig. 5. Contact angle (CA) of dried polyelectrolyte membranes. a SEBS, **b** SSBS-SN-10, **c** SSBS-SN-20, **d** SSBS-SN-30, **e** SSBS-SN-40 and **f** SEBS-IM-100. SSBS-SN-x, x is defined The percentage of grafting degree.

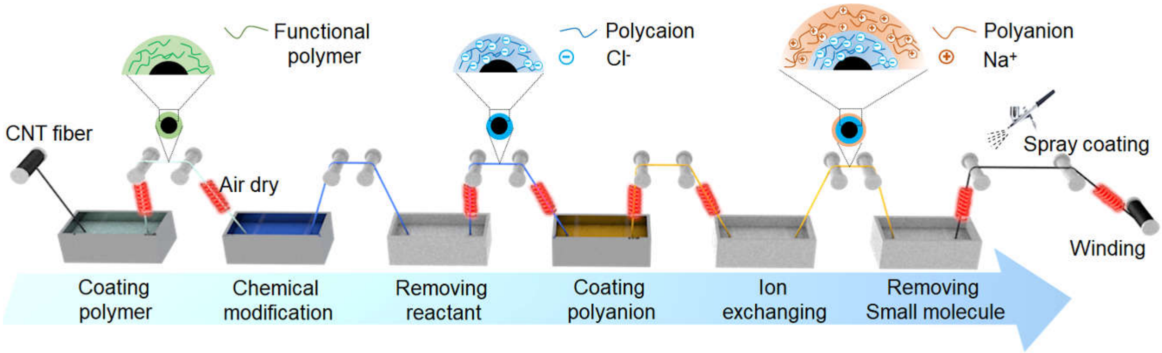

Supplementary Fig. 6. Schematic diagram of the home-made integrated fabrication instrument for ionic-junction fiber. CSEBS slurry, 1-hexylimidazole, deionized (DI) water, SSEBS slurry and NaCl solution were sequentially placed in the coating tank to carry out a series of process steps including CSEBS coating, quaternization reaction, small molecule removal, SSEBS coating and ion exchange. Fibers were dried and annealed at 120 °C in heating drums.

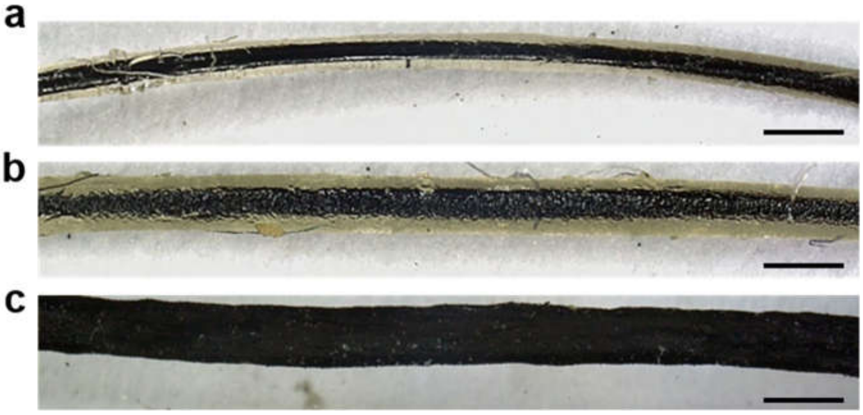

Supplementary Fig. 7. Optical photograph of the completed fiber. a CNT@SEBS-IM fiber (250-300 μm), **b** CNT@SEBS-IM@SEBS-SN fiber (400-450 μm) and **c** CNT@SEBS-IM@SEBS-SN@CNT fiber (480-520 μm). Micrographs, images representative of 3 experiments, scale bar = 500 μm

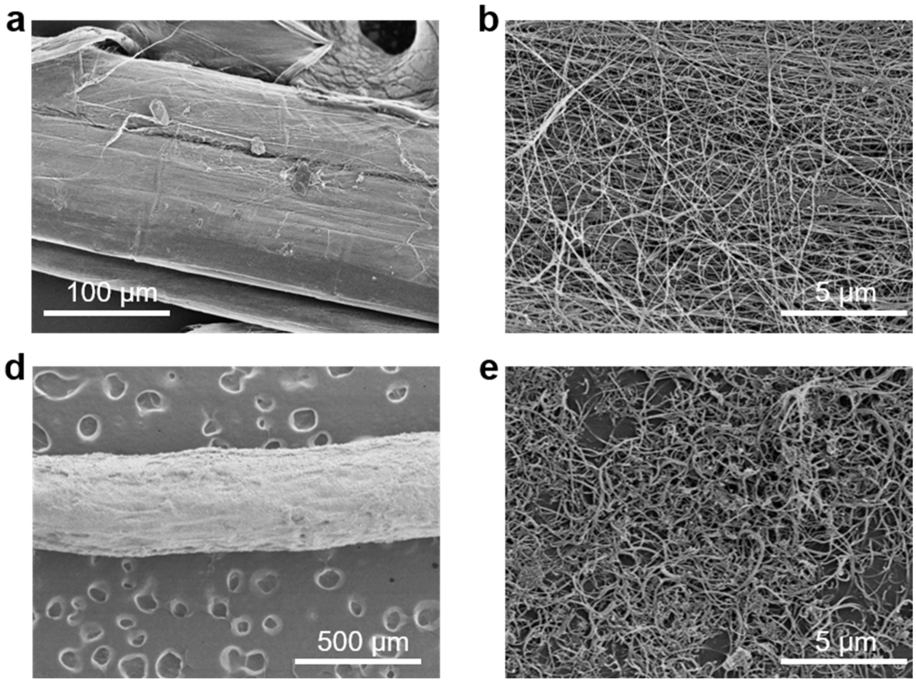

Supplementary Fig. 8. SEM images of the CNT fiber before and after surface coating. a CNT fiber (core layer) and **b** fiber under magnification; **c** CNT fiber after surface coating (sheath layer) and **d** coating under magnification. Micrographs, images representative of 3 experiments.

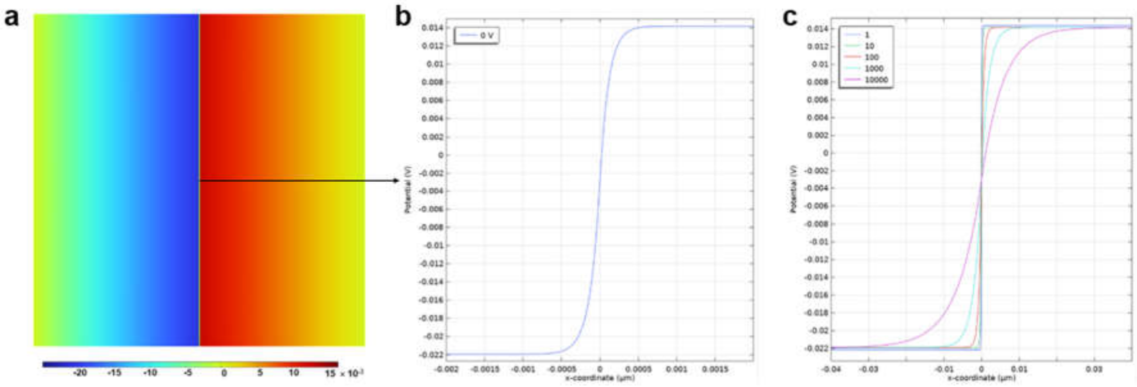

Supplementary Fig. 9. Simulated induced potential distribution profile along the thickness direction of the fiber-shaped ionic diode by at zero bias. **a** 2D image of the simulation, **b** using relative permittivity of 4, and **c** using different assumed relative permittivity values.

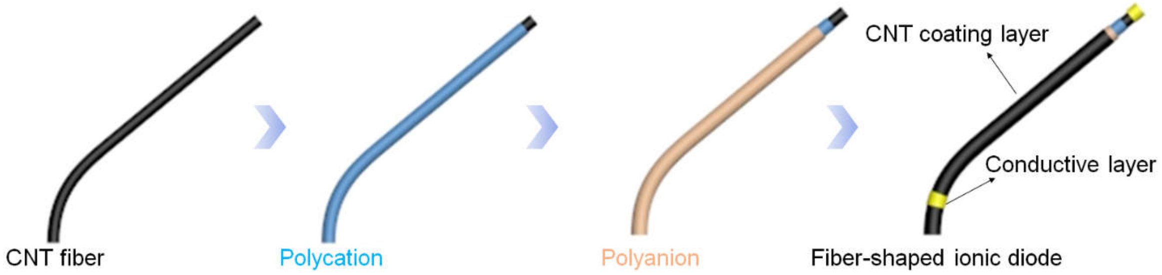

Supplementary Fig. 10. Schematic illustration of the preparation of the fiber-shaped ionic diode.

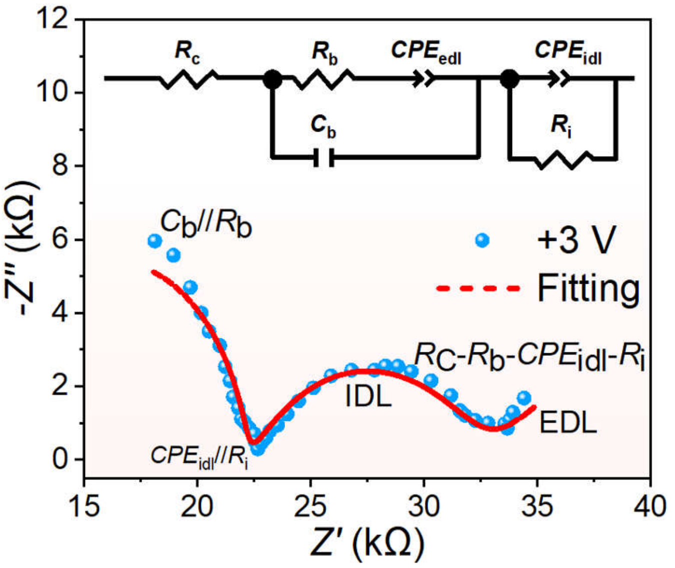

Supplementary Fig. 11. Nyquist plot of SEBS-IM / SEBS-SN under +3.0 V. (Inset, the equivalent circuit model for a SEBS-IM / SEBS-SN junction under DC bias.) In the model contains, a dielectric capacitance (C_b) is the polarization of the ions at high frequency. A resistor (R_b) is connected in parallel and it reflects the drift of free ions at a moderate frequency. Contact resistance *(R*c) is added in series with the whole circuit. At low frequency, free ions accumulate at the interface and form an EDL or IDL that can be described by a constant phase element (*CPE*). CPE_{edl} describes the EDL capacitance and CPE_{idl} describes the IDL capacitance. R_i is a resistor for interfacial ionic current. CPE_{idl} and R_i are connected in parallel, and they are added in series with SEBS-IM/SEBS-SN. The blue dotted line is the real Nyquist plot, and the red solid line is the fitting curve. Fitting parameters for the circuit components in the equivalent circuit model were listed in **Table S1**. Source data are provided as a Source Data file.

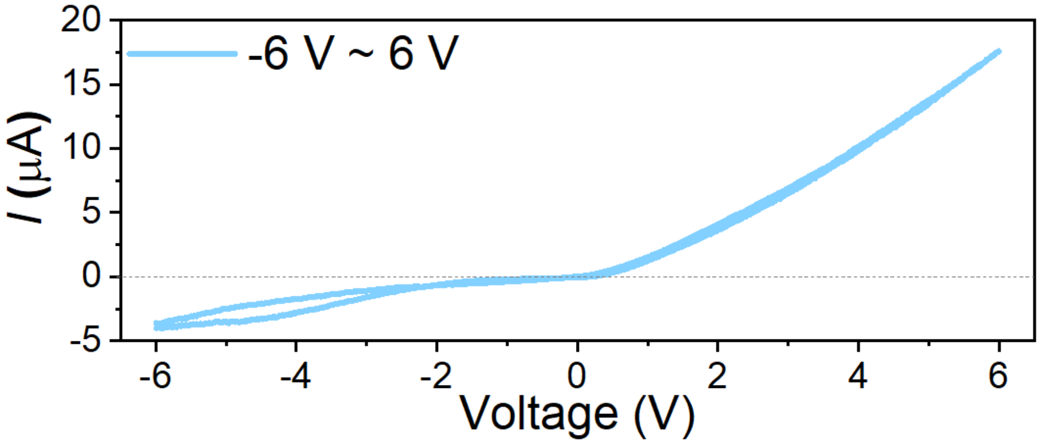

Supplementary Fig. 12. CV curves of ionic-junction fibers at different voltage ranges with a scan rate of 10 mV/s. No obvious redox peaks appear when the voltage is limited between -6 ~ +6 V, which is wider than the operation voltage window applied in our fiber-shaped intronic devices. Source data are provided as a Source Data file.

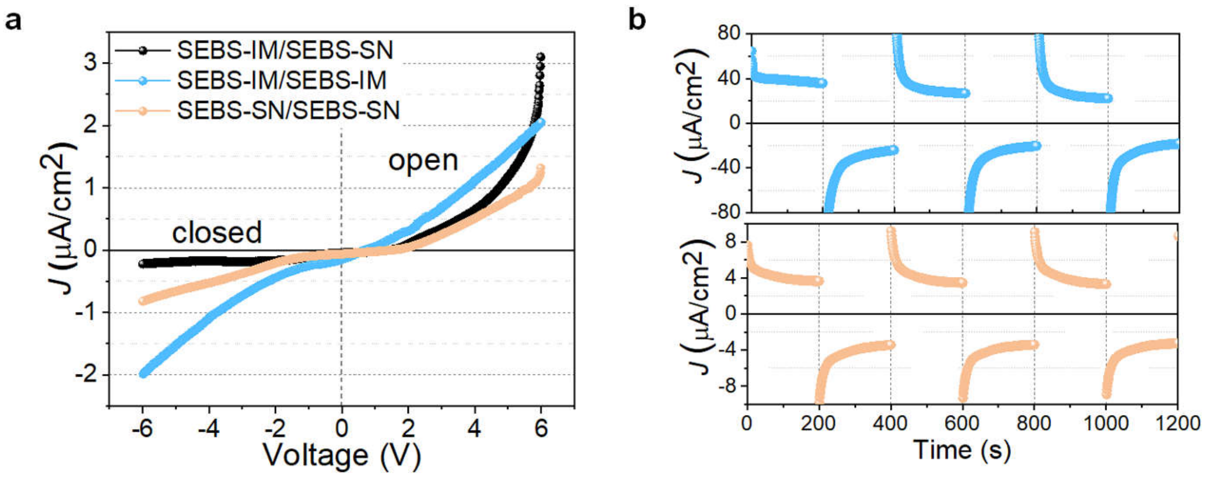

Supplementary Fig. 13. Full *J-V* characteristics of ionic polyelectrolyte junctions (SEBS-IM/SEBS-IM, SEBS-SN/SEBS-SN, and SEBS-IM/SEBS-SN). a Curves at a scan voltage from -6.0 V ~ 6.0 V. **b** Rectification of different junctions at a bias voltage of ±3.0 V (The CNT@SEBS-IM@ SEBS-IM@ CNT was a device that composed of a SEBS-IM/SEBS-IM junction; The CNT@SEBS-SN@ SEBS-SN@CNT was a device that composed of a SEBS-SN/SEBS-SN junction). Source data are provided as a Source Data file.

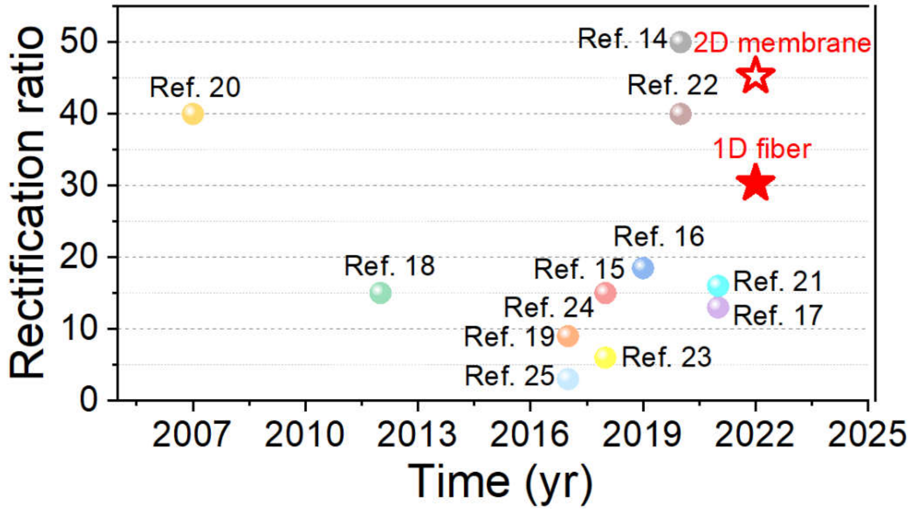

Supplementary Fig. 14. Comparison of the rectification performance of reported solid-state ionic diode devices with our fiber-shape d ionic diodes[14-25]. 1D, one-dimensional; 2D, two-dimensional. Source data are provided as a Source Data file.

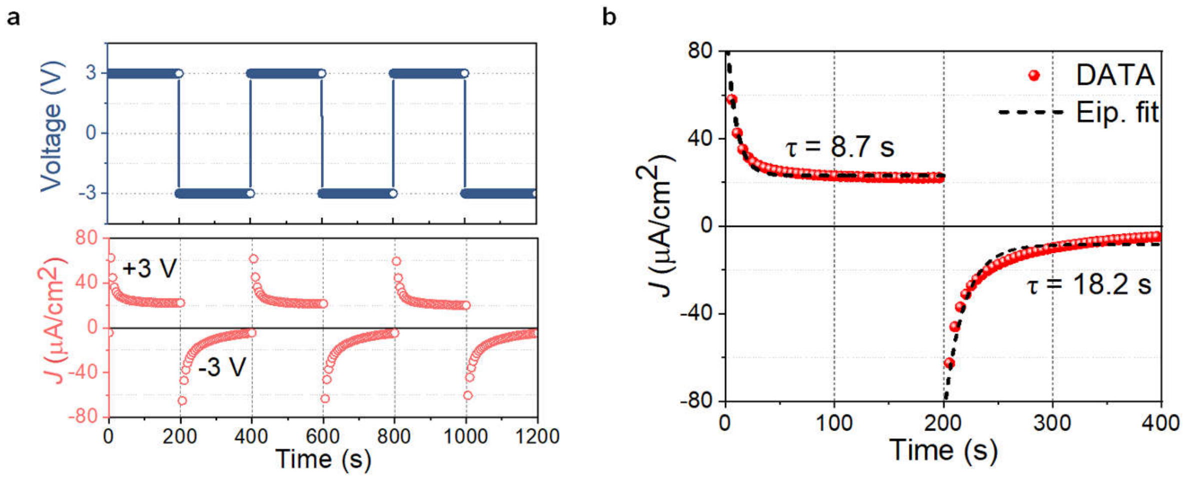

Supplementary Fig. 15. Transient response characteristics of the fiber-shaped ionic diode (Φ = 153 μm, Φ, diameter). a Rectification of fiber-shaped ionic diode under an alternating potential of ±3.0 V. (Applied voltage is plotted on the top, while the corresponding current is demonstrated on the bottom.). **b** Transient response of current density as reported in **a**. A forward bias of +3.0 V and a reverse bias of -3.0 V was applied. The black dotted line was an exponential fit with a storage time constant of 8.7 s and a transition time of 18.2 s for the switch-on in the fiber-shaped ionic diode. Source data are provided as a Source Data file.

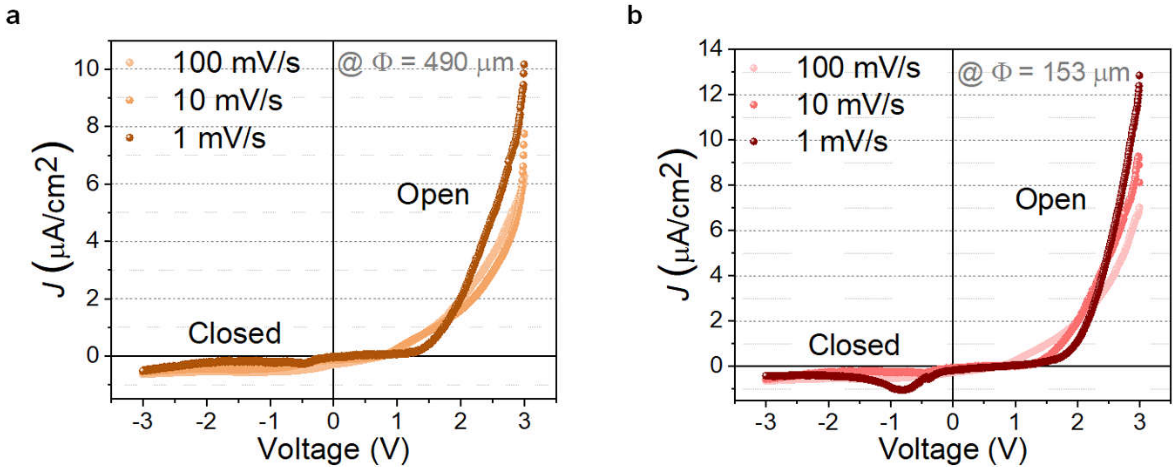

Supplementary Fig. 16. Rectification ratio of the fiber-shaped ionic diode with two diameters at different scanning rates from 100 mV s^{-1} to 1 mV s^{-1}. The diameter is **a** 490 μm and **b** 153 μm. Source data are provided as a Source Data file.

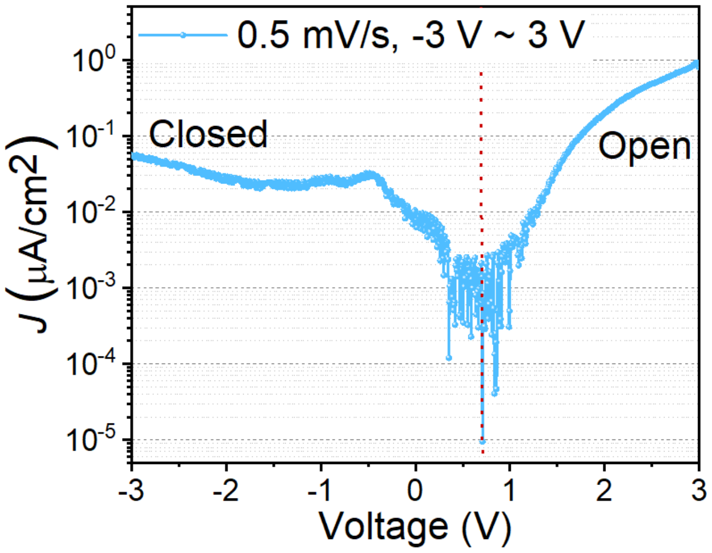

Supplementary Fig. 17. The rectifying behaviors at different scan rates of 0.5 mV/s were evaluated via linear sweep voltammetry from -3 V ~ 3 V and -6 V ~ 6 V, respectively. Source data are provided as a Source Data file.

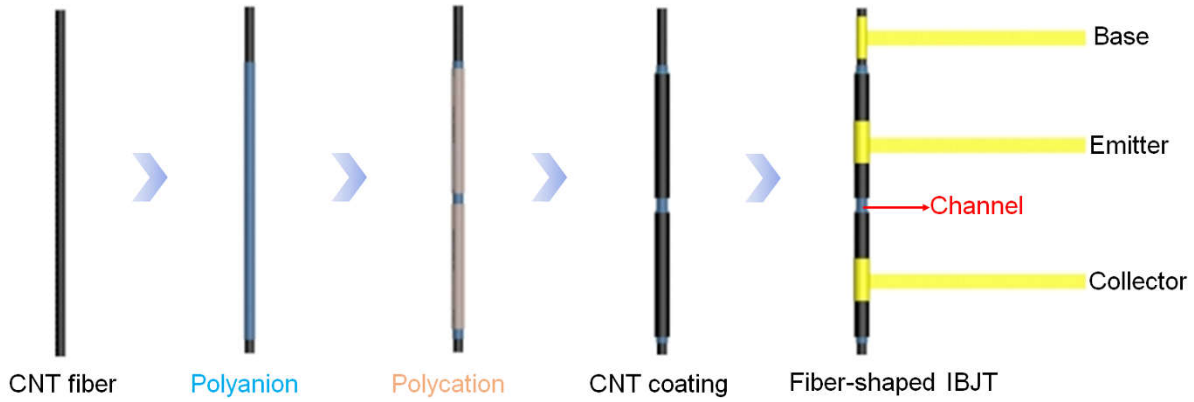

Supplementary Fig. 18. Schematic illustration of the preparation of the fiber-shaped IBJT.

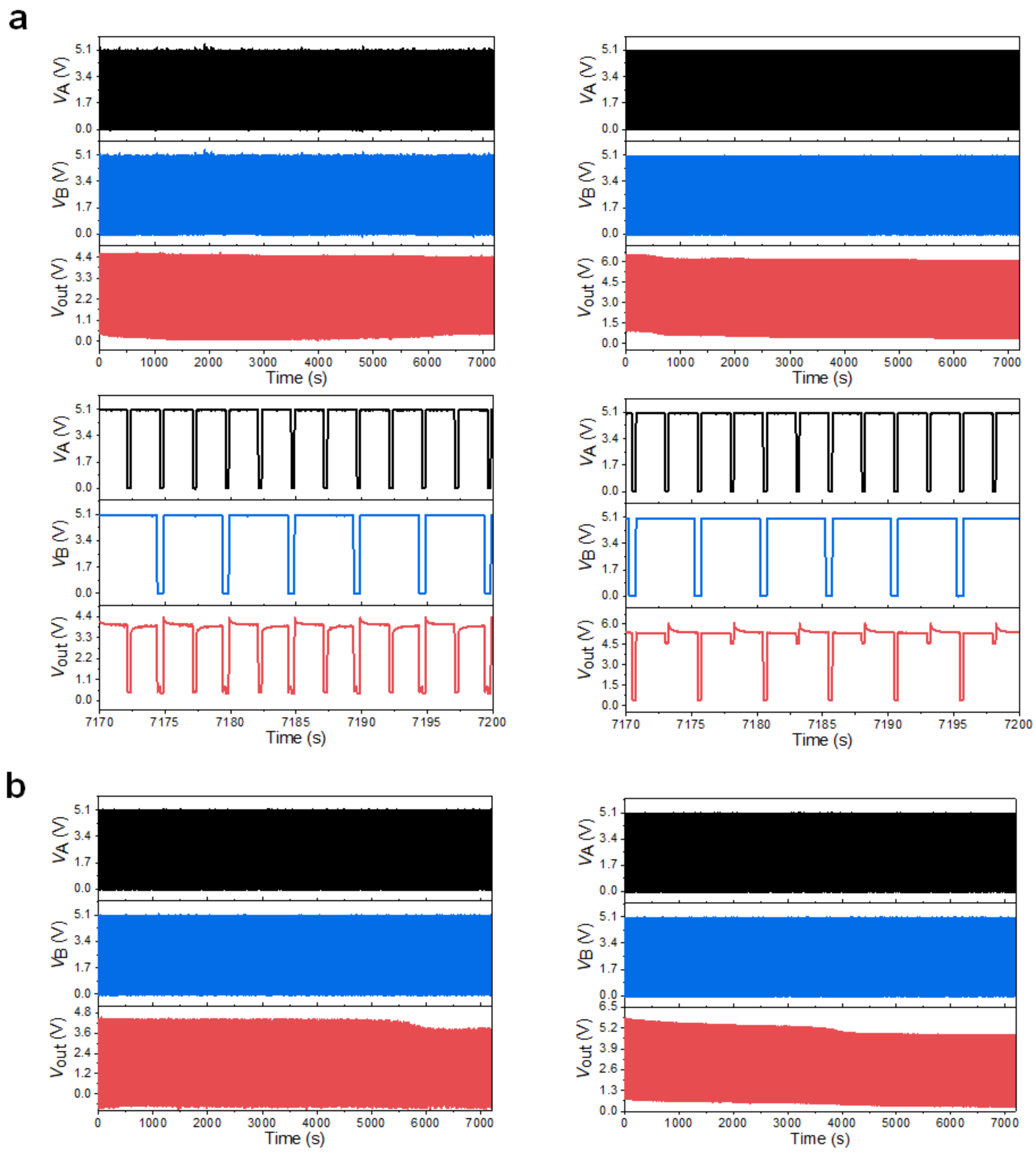

Supplementary Fig. 19. Demonstration of ionic-junction logic gates based on multiple fiber-shaped ionic diodes. Experimental datas of the input and output signals of the “AND” and the “OR”gate at **a** a 90% duty cycle square wave, as well as **b** a 50% duty cycle square wave . Source data are provided as a Source Data file.

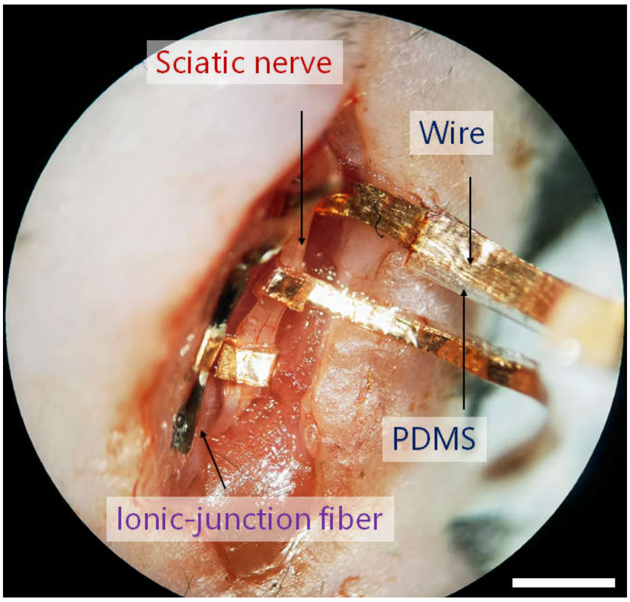

Supplementary Fig. 20. An intraoperative image of fibers effectively connect with the nerve through flexible metal electrodes in a mouse (Scale bar: 3 mm).

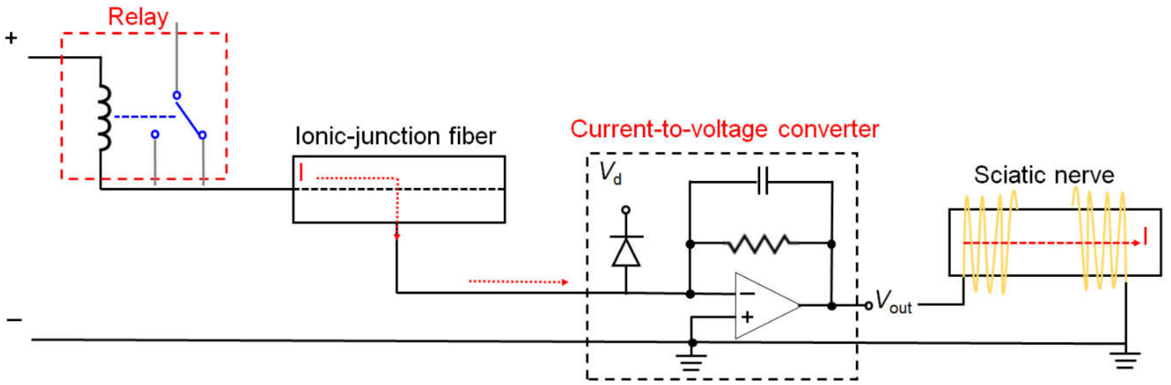

Supplementary Fig. 21. A current-to-voltage signal amplifier converter was applied between the ionic-junction fiber and the sciatic nerve[26]. A voltage of 0.5 V is amplified to stimulate biological efferent nerves and muscles to initiate movement, which results in inducing a significant movement of the hind limb. (V_{supply}: supplied voltage to the circuit; V_d: supply voltage to the amplifier; V_{out}: output voltage from amplified to nerves). The whole amplifier circuit is composed of an Arduino relay, an ionic-junction fiber, a current-to-voltage signal amplifier converter and the sciatic nerve in a rat.

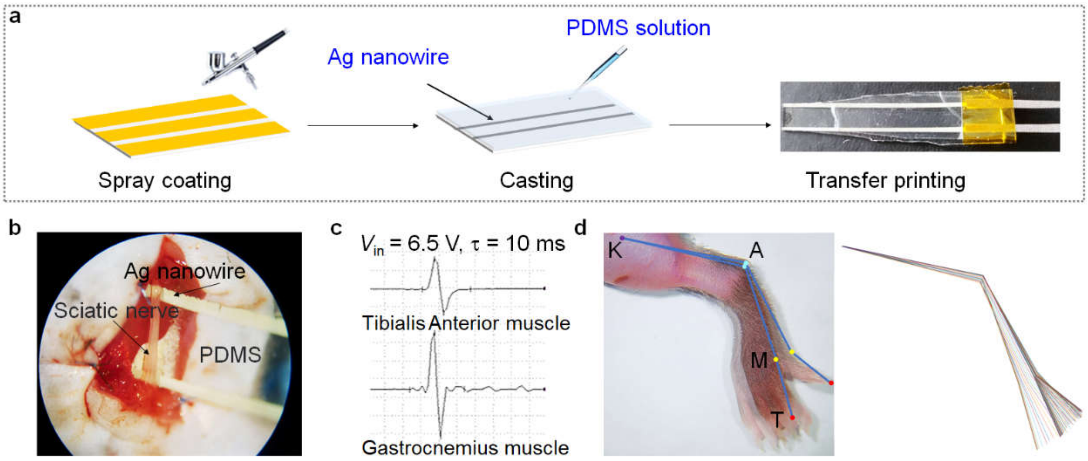

Supplementary Fig. 22. Difference of CMAPs induced by a flexible electrode connecting the fiber device. a The process of preparing flexible electrodes by transfer printing. **b** An intraoperative image of the flexible AgNWs@PDMS electrodes in close contact with the distal end of the sciatic nerve. **c** CMAPs of Gastrocnemius muscle and Tibialis Anterior muscle when applied a square wave voltage of 6.5 V with a stimulus frequency of 1 Hz and a pulse width of 10 ms when connected flexible electrodes with sciatic nerve. V_{in}, input voltage. **d** Angle analysis of extension and flexion in the ankle joint induced by a flexible electrode. The knee joint (K), ankle joint (A), metatarsophalangeal joint (M), and the end of toes (T) were labeled on the hindlimb of the mouse (Inset: Representative images of reproduced hindlimb motion trajectory elicited by electrical stimulation *via* ionic-junction fiber devices).

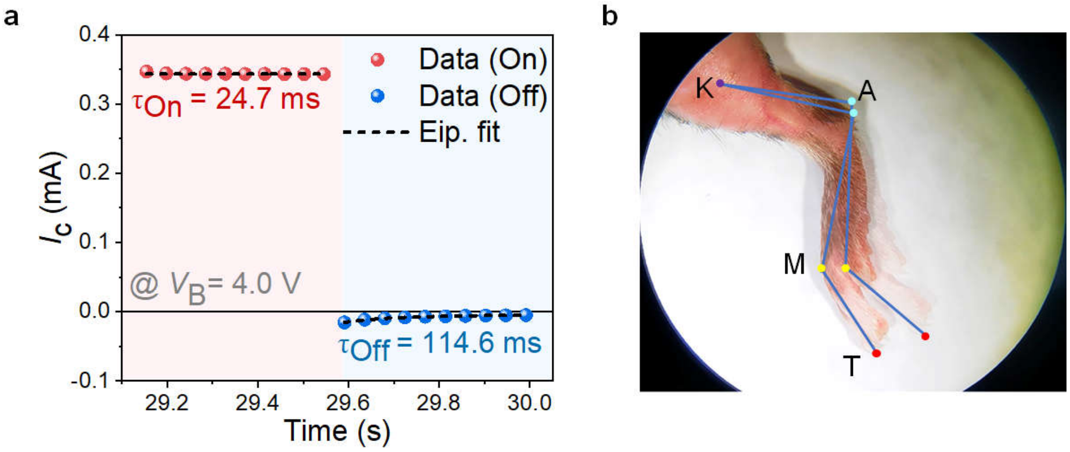

Supplementary Fig. 23. Output characteristic of signal transmission pathways made of a fiber-shaped IBJT. a Transient response of current density as reported in Fig. 5D. The black dotted line was an exponential fit with a storage time constant of 24.7 ms and a transition time of 114.6 ms for the switch-on in the fiber-shaped IBJT. **b** Angle analysis of extension and flexion in the ankle joint induced when a fiber-shaped IBJT was connected with the the sciatic nerve. The knee joint (K), ankle joint (A), metatarsophalangeal joint (M), and the end of toes (T) were labeled on the hindlimb of the mouse. Source data are provided as a Source Data file.

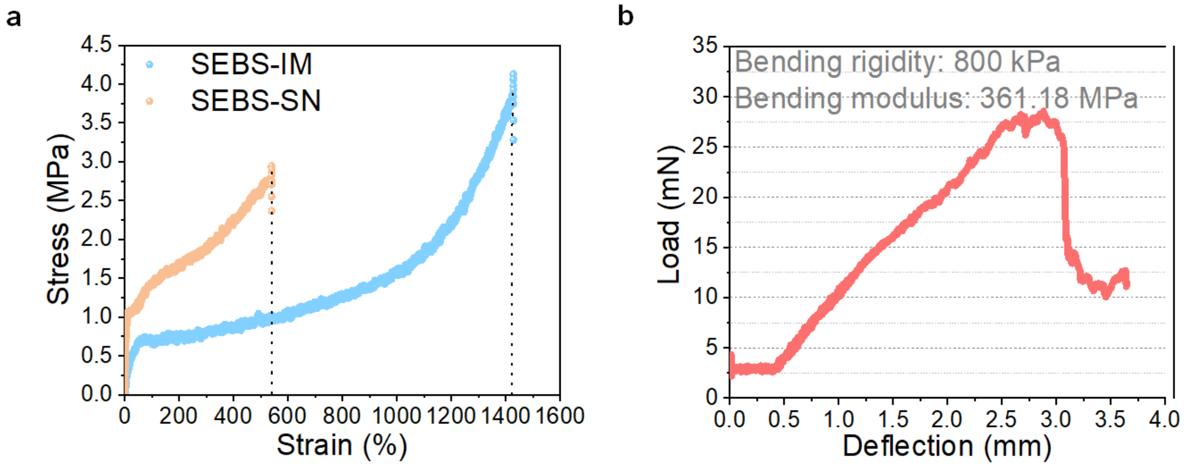

Supplementary Fig. 24. Mechanical properties of the component of the polyelectrolytes in the fiber. a Stress-strain curves of SEBS-IM and SEBS-SN, along with the elongations at break of ~1410 % for SEBS-IM and ~530% for SEBS-SN. **b** The softness of the ionic-junction fibers, characterized by bending stiffness. Source data are provided as a Source Data file.

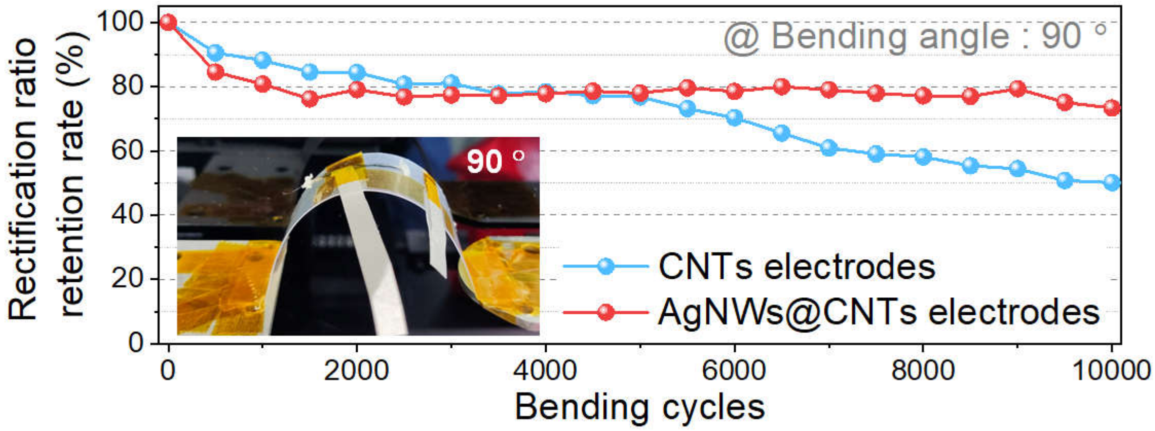

Supplementary Fig. 25. Comparison of rectification retention performance of fibers with CNT electrodes and with CNT@AgNWs electrodes under cyclic bending. AgNWs, silver nanowires. Source data are provided as a Source Data file.

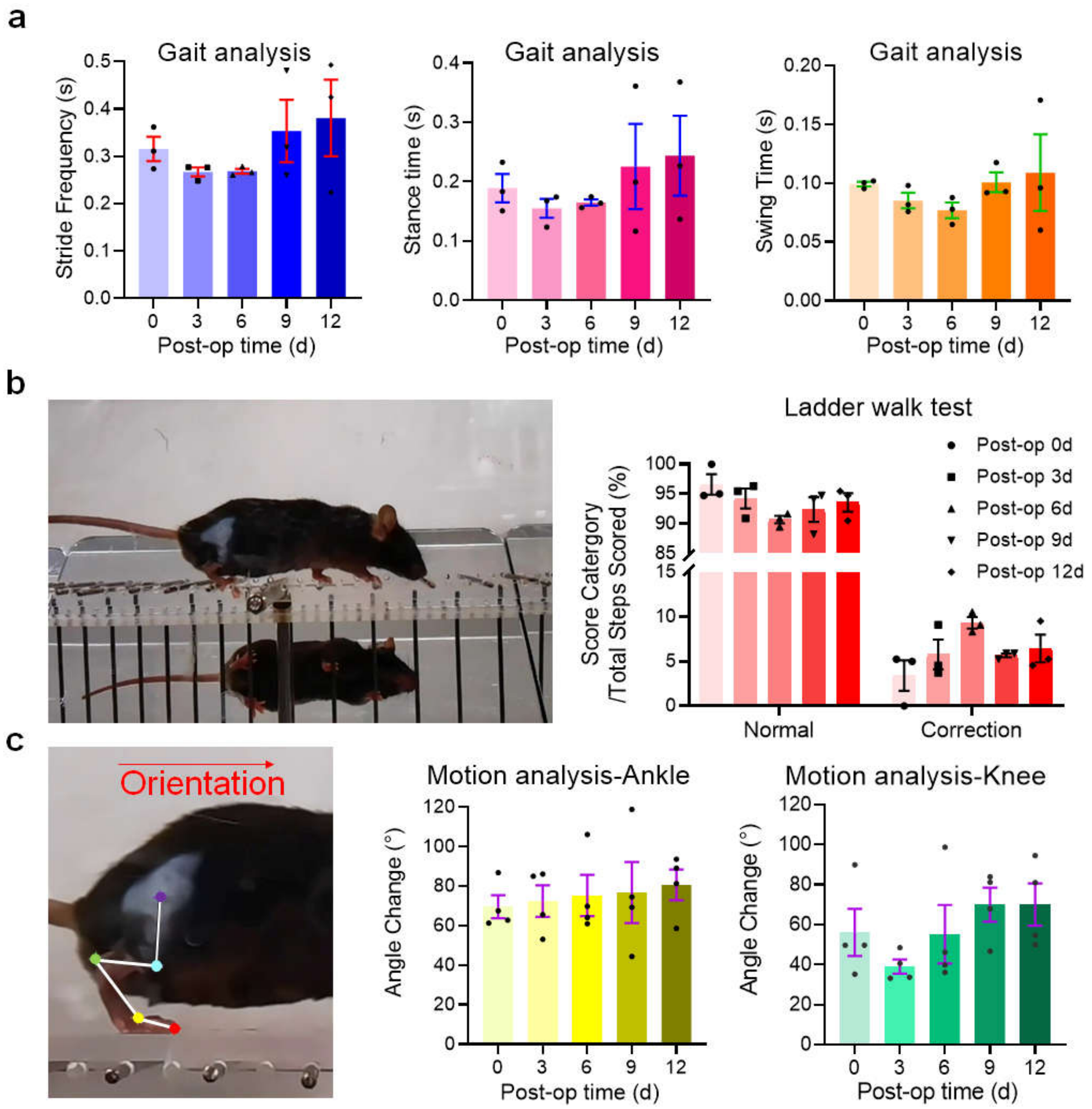

Supplementary Fig. 26. Assessment of locomotor activity during Gait analysis, ladder rung walking test and motion analysis. a The stride frequency, stance time and swing time was investigated in a mouse before and at different time points after implantion operation. Data were presented as mean ± s.e.m. (n=3). **b** Representative photograph of Ladder rung walking test was shown(left) and the percentage of "Normal" and "Correction" score category/total steps scored (right) was calculated to reflect the function of sciatic nerve. Data were presented as mean ± s.e.m. (n=3). **c** The spontaneous movement of hindlimb was reducible to the movement of the five points: hip (purple), knee (blue), ankle (green), metatarsophalangeal joint (yellow) and toes(red). The maximum angle change of ankle (left) and knee joints were analyzed to show the spontaneous movement capacity of hindlimb joints (right). Data were presented as mean ± s.e.m. (n=4). Source data are provided as a Source Data file.

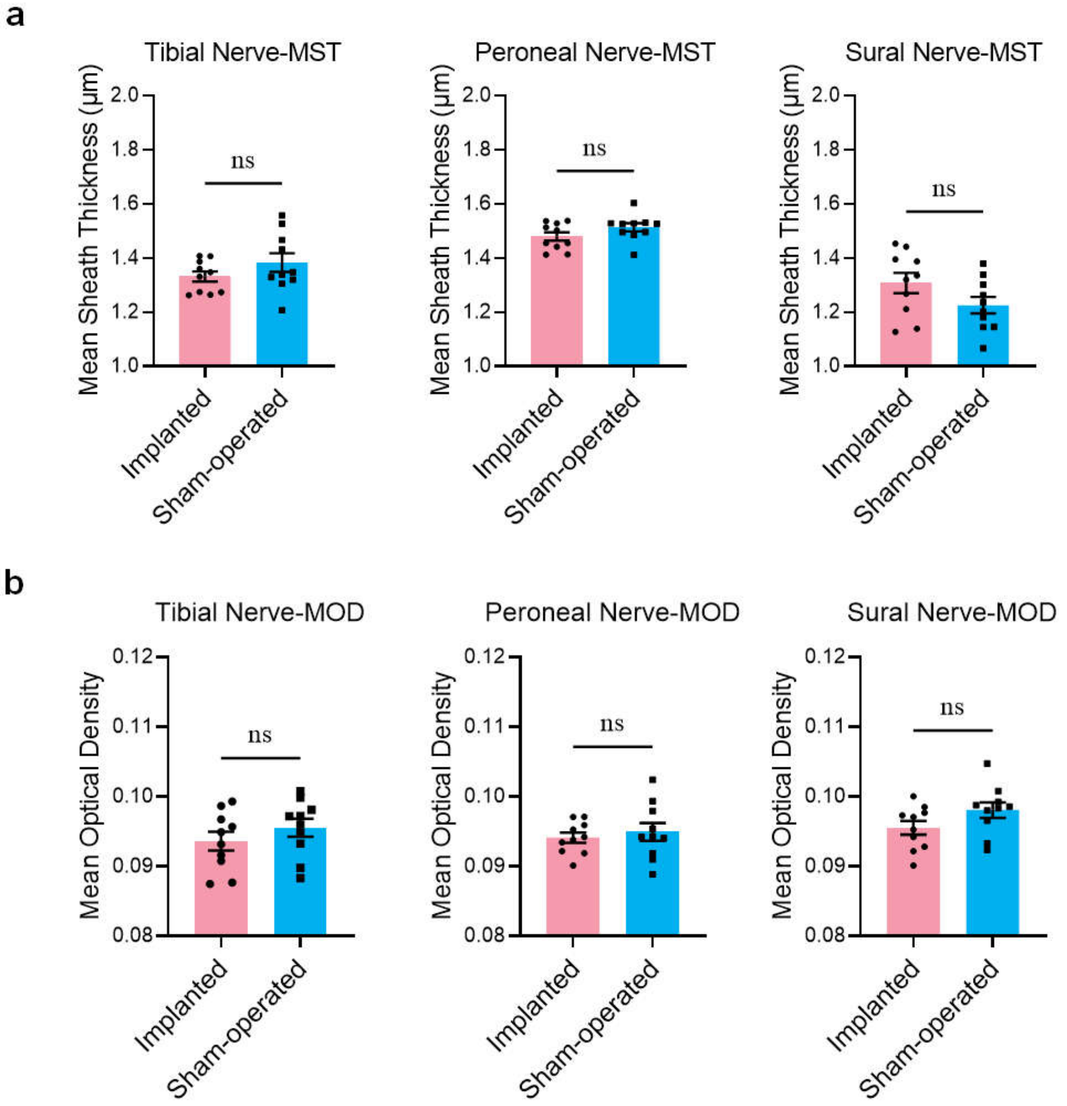

Supplementary Fig. 27. Quantitative analysis of mean sheath thickness (MST) and mean optical density (MOD) of sciatic nerve myelin sheath. Data are shown as mean ± s.e.m. (n = 10). Two-sided unpaired t test was used for the comparison between the two groups after normal distribution was confirmed using the Kolmogorov-Smirnov normality test. Source data are provided as a Source Data file.

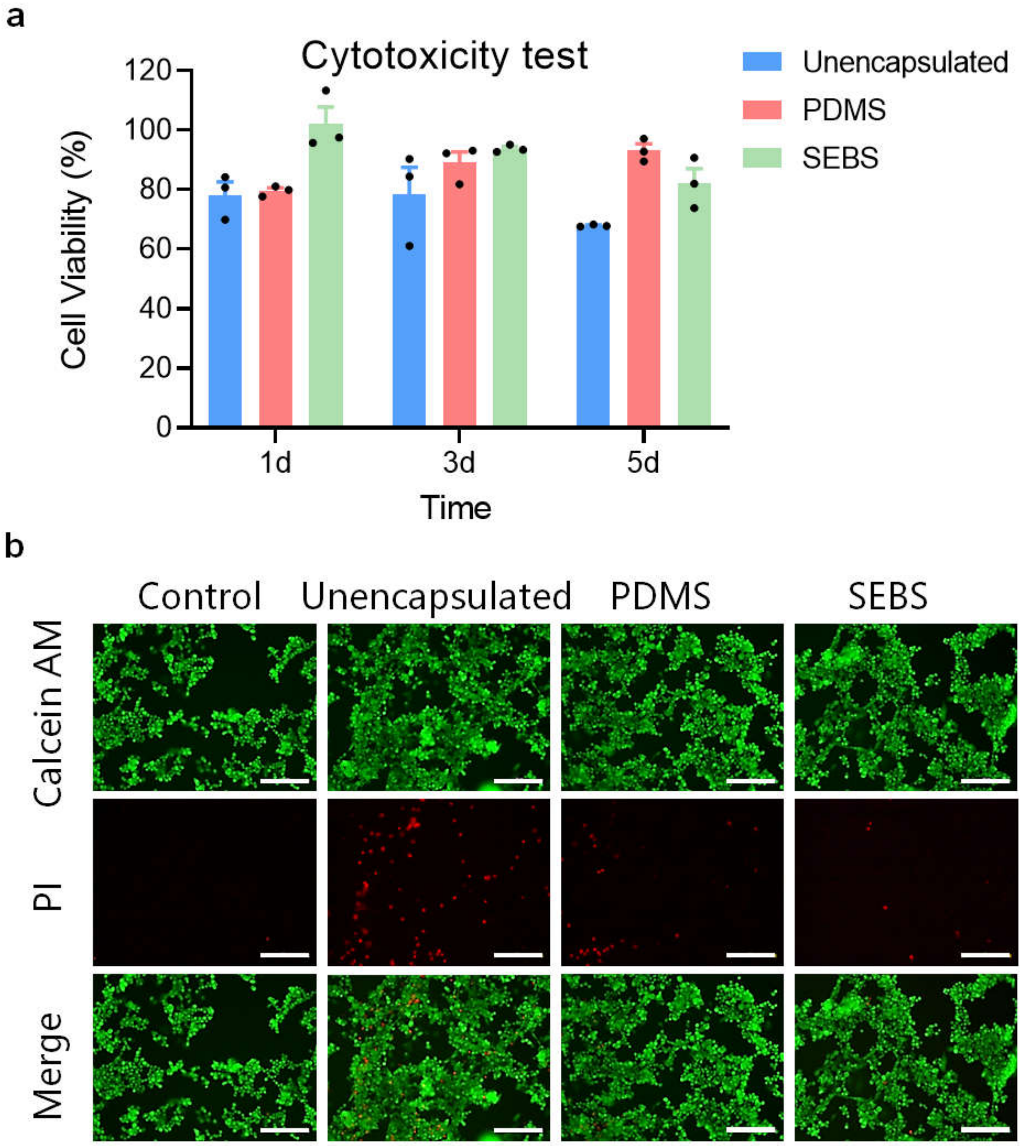

Supplementary Fig. 28. Cytotoxicity of ionic-junction fiber. a Quantitative CCK-8 assay after co-incubation for 1, 3, and 5 days, respectively. CCK-8, Cell Counting Kit-8. **b** Live/Dead staining of PC12 cells after co-incubation with ionic-junction fiber (scale bar = 25 μm). All data are expressed as means ± s.e.m. (n = 3). Source data are provided as a Source Data file.

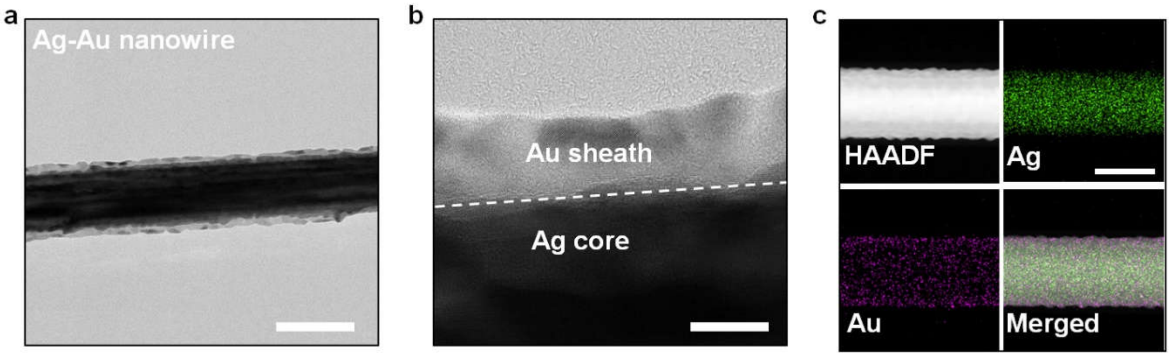

Supplementary Fig. 29. Characterization of the Ag-Au nanowire. a TEM image of the Ag-Au core-sheath nanowire. Scale bar, 50 nm. **b** The dashed line indicates the boundary between the Ag core and Au sheath. Scale bar, 5 nm. **c** Energy Dispersive Spectrometer (EDS) elemental mapping of Ag and Au in the Ag-Au nanowire, and a merged image confirming the core-sheath structure. Micrographs, images representative of 3 experiments, scale bar = 100 nm.

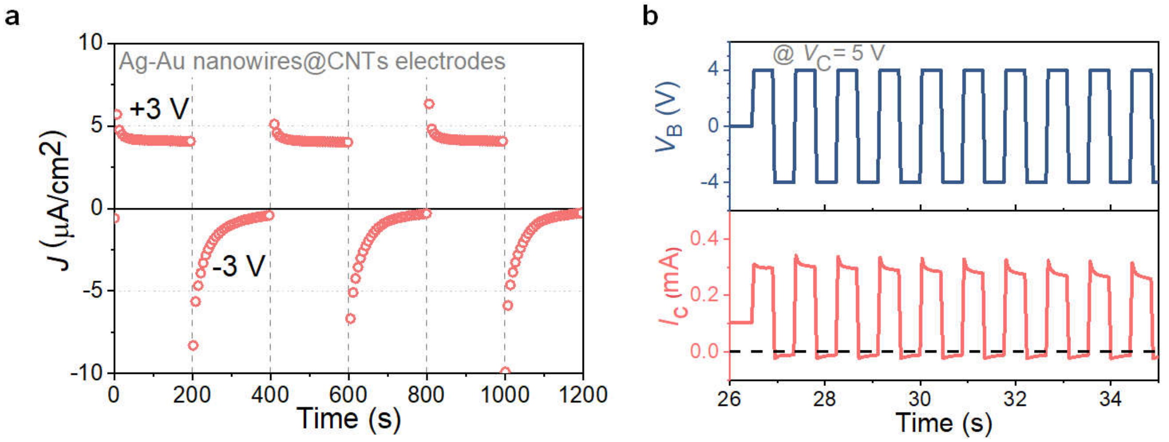

Supplementary Fig. 30. Output signals of two artificial nerve pathway that constructed with Ag-Au nanowires. a Current-voltage (*I-V*) rectification behaviour of the fiber-shaped ionic diode. **b** Output characteristic (I_C-V_C) curves of fiber-shaped IBJT. Source data are provided as a Source Data file.

Supplementary Table 1. Fitting parameters for circuit components in the equivalent circuit model under a forward bias of +3.0 V.

Element	R_c (kΩ)	R_b (kΩ)	C_b (nF)	CPE_{idl}	CPE_{edl}	R_i (kΩ)
SEBS-IM / SEBS-SN (+ 3.0 V)	10.36 ± 0.46	11.64 ± 0.43	98.42 ± 8.13	0.63 ± 0.03	0.36 ± 0.13	10.14 ± 0.97

Reference

1. Dai, P., Mo, Z. H., Xu, R. W., Zhang, S. & Wu, Y. X. Cross-linked quaternized poly(styrene-b-(ethylene-co-butylene)-b-styrene) for anion exchange membrane: synthesis, characterization and properties. *ACS Appl. Mater. Interfaces* **8**, 20329-20341 (2016).
2. Sood, R., Donnadio, A., Giancola, S., Kreisz, A., Jones, D. J. & Cavaliere, S. 1,2,3-Triazole-functionalized polysulfone synthesis through microwave-assisted copper-catalyzed click chemistry: a highly proton conducting high temperature membrane. *ACS Appl. Mater. Interfaces* **8**, 16897-16906 (2016).
3. Hwang, H. Y., Koh, H. C., Rhim, J. W. & Nam, S. Y. Preparation of sulfonated SEBS block copolymer membranes and their permeation properties. *Desalination* **233**, 173-182 (2008).
4. He, J. et al. Scalable production of high-performing woven lithium-ion fibre batteries. *Nature* **597**, 57-63 (2021).
5. Wang, Y. et al. Direct graphene-carbon nanotube composite ink writing all-solid-state flexible microsupercapacitors with high areal energy density. *Adv. Funct. Mater.* **30**, 1907284 (2020).
6. Qu´er´e, D. Fluid coating on a fiber. *Annu. Rev. Fluid Mech.* **31**, 347-384 (1999).
7. Austelle, C. W., *et al.* A comprehensive review of vagus nerve stimulation for depression. *Neuromodulation* **25**, 309-315 (2022).
8. Olofsson, P. S., *et al.* Single-pulse and unidirectional electrical activation of the cervical vagus nerve reduces tumor necrosis factor in endotoxemia. *Bioelectron. Med.* **2**, 37-42 (2015).
9. Metz GA, Whishaw IQ. Cortical and subcortical lesions impair skilled walking in the ladder rung walking test: a new task to evaluate fore- and hindlimb stepping, placing, and co-ordination. *J Neurosci Methods*. 115(2):169-179 (2002).
10. Wang H, Spinner RJ, Windebank AJ. Quantitative evaluation of movement and strength of the upper limb after transection of the C-7 nerve: is it possible in an animal model?. *J Neurosurg Spine* 10(2):102-110 (2009).
11. Clarke KA, Still J. Gait analysis in the mouse. *Physiol Behav*. 66(5):723-729. (1999).
12. Kodým, R., Šnita, D. & Bouzek, K. Mathematical Modeling of Electromembrane Processes. In: *Current Trends and Future Developments on (Bio-) Membranes*) (Elsevier, Amsterdam, 2019).
13. Butkewitsch, S. & Scheinbeim, J. Dielectric properties of a hydrated sulfonated poly(styrene-ethylene/butylenes-styrene) triblock copolymer. *Appl. Surf. Sci.* **252**, 8277-8286 (2006).

14. Lee, H.-R., *et al.* A stretchable ionic diode from copolyelectrolyte hydrogels with methacrylated polysaccharides. *Adv. Funct. Mater.* **29**, 1806909 (2019).
15. Wang, Y., Wang, Z., Su, Z. & Cai, S. Stretchable and transparent ionic diode and logic gates. *Extreme Mech. Lett.* **28**, 81-86 (2019).
16. Hou, Y., *et al.* Flexible ionic diodes for low-frequency mechanical energy harvesting. *Adv. Energy Mater.* **7**, 1601983 (2017).
17. Kim, H. J., Chen, B., Suo, Z. & Hayward, R. C. Ionoelastomer junctions between polymer networks of fixed anions and cations. *Science* **367**, 773-776 (2020).
18. Zhao, Y., Dai, S., Chu, Y., Wu, X., Huang, J. A flexible ionic synaptic device and diode-based aqueous ion sensor utilizing asymmetric polyelectrolyte distribution. *Chem Commun.* **54**, 8186-8189 (2018).
19. Zhou, Y., *et al.* Biocompatible and flexible hydrogel diode-based mechanical energy harvesting. *Adv. Mater. Technolo.* **2**, 1700118 (2017).
20. Zhang W, Zhang, X, Lu C, Wang Y, Deng Y. Flexible and transparent paper-based ionic diode fabricated from oppositely charged microfibrillated cellulose. *J. Phys. Chem. C.* **116**, 9227-9234 (2012).
21. Zhang Y*, et al.* Hydrogel ionic diodes toward harvesting ultralow-frequency mechanical energy. *Adv. Mater.* **33**, 2103056 (2021).
22. Han, S. H., *et al.* Hydrogel-based iontronics on a polydimethylsiloxane microchip. *ACS Appl. Mater. Interfaces* **13**, 6606-6614 (2021).
23. Nyamayaro, K., *et al.* Toward biodegradable electronics: ionic diodes based on a cellulose nanocrystal-agarose hydrogel. *ACS Appl. Mater. Interfaces* **12**, 52182-52191 (2020).
24. Feng, X., *et al.* All carbon materials pn diode. *Nat. Commun.* **9**, 3750 (2018).
25. Cayre, O. J., Chang, S. T. & Velev, O. D. Polyelectrolyte diode: nonlinear current response of a junction between aqueous ionic gels. J. *J. Am. Chem. Soc.* **129**, 10801-10806 (2007).
26. Kim, Y. et al. A bioinspired flexible organic artificial afferent nerve. *Science* **360**, 998-1003 (2018).
