# Supplementary material for: Integrated opposite charge grafting induced ionic-junction fiber
Source: Nat Commun. 2023 Apr 24;14:2355. doi: 10.1038/s41467-023-37884-0 (PMC10126126; doi:10.1038/s41467-023-37884-0)
Supplement: Supplementary file 2 — Description of Additional Supplementary Files [file 41467_2023_37884_MOESM2_ESM.docx]

**Description of Additional Supplementary Files**

**File Name: Supplementary Movie 1
Description:** Integrated opposite charge grafting fabrication technique of ionic-junction fibers.

**File Name: Supplementary Movie 2
Description:** The movements of the hindlimb induced via a stimulating voltage (4.0 V) with a stimulus pulse width of 10 ms when an ionic-junction fiber was implanted inside a mouse.

**File Name: Supplementary Movie 3**

**Description:** The movements of the hindlimb induced via a current-tovoltage signal amplifier converter between the ionic-junction fiber and the sciatic nerve.

**File Name: Supplementary Movie 4**

**Description:** The movements of the hindlimb induced via a stimulating voltage (6.5 V) with a stimulus pulse width of 10 ms by using a flexible AgNWs@PDMS electrodes.

**File Name: Supplementary Movie 5**

**Description:** The movements of the hindlimb induced via a forward voltage (+4.0 V) and a reverse voltage (-4.0 V) with a stimulus pulse width of 10 ms by connecting with a fiber-shaped IBJT, respectively.

**File Name: Supplementary Movie 6**

**Description:** Ladder rung walking test of a mouse when a ionic-junction fiber was implanted inside it.
