# Supplementary material for: Integrated opposite charge grafting induced ionic-junction fiber
Source: Nat Commun. 2023 Apr 24;14:2355. doi: 10.1038/s41467-023-37884-0 (PMC10126126; doi:10.1038/s41467-023-37884-0)
Supplement: Supplementary file 9 — Reporting Summary [file 41467_2023_37884_MOESM9_ESM.pdf]

nature portfolio

Corresponding author(s): Gang Wang

Last updated by author(s): Mar 22, 2023

Reporting Summary

Nature Portfolio wishes to improve the reproducibility of the work that we publish. This form provides structure for consistency and transparency in reporting. For further information on Nature Portfolio policies, see our Editorial Policies and the Editorial Policy Checklist.

Statistics

For all statistical analyses, confirm that the following items are present in the figure legend, table legend, main text, or Methods section.

n/a	Confirmed	
☐	☒	The exact sample size (*n*) for each experimental group/condition, given as a discrete number and unit of measurement
☐	☒	A statement on whether measurements were taken from distinct samples or whether the same sample was measured repeatedly
☐	☒	The statistical test(s) used AND whether they are one- or two-sided<br>*Only common tests should be described solely by name; describe more complex techniques in the Methods section.*
☒	☐	A description of all covariates tested
☒	☐	A description of any assumptions or corrections, such as tests of normality and adjustment for multiple comparisons
☐	☒	A full description of the statistical parameters including central tendency (e.g. means) or other basic estimates (e.g. regression coefficient) AND variation (e.g. standard deviation) or associated estimates of uncertainty (e.g. confidence intervals)
☐	☒	For null hypothesis testing, the test statistic (e.g. *F*, *t*, *r*) with confidence intervals, effect sizes, degrees of freedom and *P* value noted<br>*Give P values as exact values whenever suitable.*
☒	☐	For Bayesian analysis, information on the choice of priors and Markov chain Monte Carlo settings
☒	☐	For hierarchical and complex designs, identification of the appropriate level for tests and full reporting of outcomes
☒	☐	Estimates of effect sizes (e.g. Cohen's *d*, Pearson's *r*), indicating how they were calculated

Our web collection on statistics for biologists contains articles on many of the points above.

Software and code

Policy information about availability of computer code

Data collection	Methods of material characterization, electrochemical testing and mechanical property testing data collection are summarized in the main text and supplementary materials.<br>Adobe PR 2020 was used to edit the movies; In vivo electrophysiology data was collected by Medtronic KeyPoint Portable EMG device;<br>Photographs for live/dead cell staining was collected by Nikon Ti2 fluorescence microscope.<br>The trajectory of the hindlimb was traced with Deeplabcut Toolbox 2.2.1.<br>Reproduced hindlimb motion trajectory was drawn by MATLAB 2021a.<br>Gait analysis Data was collected by Gaitlab Version1.1.<br>Figure.5a was created with Biorender.com.<br>Nikon Eclipse C1 Confocal Microscopy was used for the photographs of immunofluorescence staining.
Data analysis	The figures are drawn in Origin 2018, GraphPad Prism 9, Microsoft Excel 2019.

For manuscripts utilizing custom algorithms or software that are central to the research but not yet described in published literature, software must be made available to editors and reviewers. We strongly encourage code deposition in a community repository (e.g. GitHub). See the Nature Portfolio guidelines for submitting code & software for further information.

Data

Policy information about availability of data

All manuscripts must include a data availability statement. This statement should provide the following information, where applicable:

- Accession codes, unique identifiers, or web links for publicly available datasets
- A description of any restrictions on data availability
- For clinical datasets or third party data, please ensure that the statement adheres to our policy

All the data generated or analysed during this study are included in this published article and its Supplementary Information. The data that support the graphs within this paper and other findings of this study are available from the corresponding author upon reasonable request.

Human research participants

Policy information about studies involving human research participants and Sex and Gender in Research.

Reporting on sex and gender	N/A
Population characteristics	N/A
Recruitment	N/A
Ethics oversight	N/A

Note that full information on the approval of the study protocol must also be provided in the manuscript.

Field-specific reporting

Please select the one below that is the best fit for your research. If you are not sure, read the appropriate sections before making your selection.

☒ Life sciences ☐ Behavioural & social sciences ☐ Ecological, evolutionary & environmental sciences

For a reference copy of the document with all sections, see nature.com/documents/nr-reporting-summary-flat.pdf

Life sciences study design

All studies must disclose on these points even when the disclosure is negative.

Sample size	The sciatic nerves of 3 mice on left side were exposed and stimulated for the measurement of action angle change in Fig.5f;<br>2 IBJT devices were implanted close to left sciatic nerve of 2 mice, and the sham-operated right sciatic nerves were used as control in Fig.5g;<br>Quantitative analysis of MST and MOD for each group were performed in 2 slice, and 5 areas of same size were sampled for statistical analysis in each slice in Fig.S26;<br>3 mice after IBJT implantation were used for gait analysis and ladder walk test in Fig.S27a-b;<br>4 mice after IBJT implantation were used for Motion analysis in Fig.S27c;<br>3 well for each group was used for cytotoxicity test of PC12 cells in Fig.S28a.<br>Another 3 well for each group was used for Live/Dead staining of PC12 cells in Fig.S28b.
Data exclusions	No data was excluded from the analyses.
Replication	All the results can be replicated because they were only recorded if similar results were observed repeatedly for 3 times.
Randomization	All the results are randomly recorded in all the experiments.
Blinding	The researchers were blind to the experimental grouping in immunofluorescent staining test of sciatic nerve, PC12 Cytotoxicity test and PC12 Live/Dead staining test.

Reporting for specific materials, systems and methods

We require information from authors about some types of materials, experimental systems and methods used in many studies. Here, indicate whether each material, system or method listed is relevant to your study. If you are not sure if a list item applies to your research, read the appropriate section before selecting a response.

Materials & experimental systems

n/a	Involved in the study	
☐	☒	Antibodies
☐	☒	Eukaryotic cell lines
☒	☐	Palaeontology and archaeology
☐	☒	Animals and other organisms
☒	☐	Clinical data
☒	☐	Dual use research of concern

Methods

n/a	Involved in the study	
☒	☐	ChIP-seq
☒	☐	Flow cytometry
☒	☐	MRI-based neuroimaging

Antibodies

Antibodies used

Primary Antibodies:
Anti-iba1 antibody(MABN92) was purchased from Sigma-Aldrich, US.
Anti-MBP antibody(NB600-717) was purchased from Novus Biologicals, US.
Secondary antibodies:
Alexa Fluor 488-conjugated Goat Anti-Mouse IgG (H+L) (GB25301)was purchased from Servicebio,China.
Cy3-conjugated Goat Anti-Rat IgG (H+L)(GB21302) was purchased from Servicebio,China.

Validation

Validation details of the primary antibodies are available on the manufacturers' websites and the related references:
Anti-iba1 antibody, Cat.# MABN92, https://www.sigmaaldrich.cn/CN/zh/product/mm/mabn92
Anti-MBP antibody, Cat.# NB600-717, https://www.novusbio.com/products/mbp-antibody-12_nb600-717#PublicationSection
Alexa Fluor 488-conjugated Goat Anti-Mouse IgG (H+L),Cat.# GB25301, https://www.servicebio.cn/goodsdetail?id=272
Cy3-conjugated Goat Anti-Rat IgG (H+L),Cat.# GB21302, https://www.servicebio.cn/goodsdetail?id=252

Eukaryotic cell lines

Policy information about cell lines and Sex and Gender in Research

Cell line source(s)

The PC-12 cell line (SCSP-517)were kindly provided by Stem Cell Bank, Chinese Academy of Sciences.

Authentication

The PC-12 cell line were authenticated by morphology, which was was consistent with that described in the instructions the Stem Cell Bank, Chinese Academy of Sciences provided.

Mycoplasma contamination

The PC-12 cell line were tested negative for mycoplasma contamination by Stem Cell Bank, Chinese Academy of Sciences.

Commonly misidentified lines
(See ICLAC register)

No commonly misidentified cell lines were used in the study.

Animals and other research organisms

Policy information about studies involving animals; ARRIVE guidelines recommended for reporting animal research, and Sex and Gender in Research

Laboratory animals

Male C57BL/6 mice of 8-10 weeks were obtained from the Shanghai Laboratory Animal Center, CAS (Shanghai, China).

Wild animals

No wild animals were used in the study.

Reporting on sex

Only male C57BL/6 mice were used as model animals in all the animal researches.

Field-collected samples

No field collected samples were used in the study.

Ethics oversight

Animal care and experiments were performed in accordance with the National Institutes of Health Guide for the Care and Use of Laboratory Animals and were approved by the Animal Care and Use Committee of Shanghai Medical College of Fudan University.

Note that full information on the approval of the study protocol must also be provided in the manuscript.
